# Supplementary material for: Generation and trapping of a mesoderm biased state of human pluripotency
Source: Nat Commun. 2020 Oct 5;11:4989. doi: 10.1038/s41467-020-18727-8 (PMC7536399; doi:10.1038/s41467-020-18727-8)
Supplement: Supplementary file 1 — Supplementary Information [file 41467_2020_18727_MOESM1_ESM.pdf]

Generation and Trapping of a Mesoderm Biased State of Human Pluripotency

Stavish et al.

SFig 1

a

	FPKM Values						LOG2 Fold Change from M(-)3(+)	
	MIXL1 -GFP(-)/ SSEA3(+)		*MIXL1* -GFP(+)/ SSEA3(+)		*MIXL1* -GFP(+)/ SSEA3(-)		*MIXL1* -GFP(+)/ SSEA3(+)	*MIXL1* -GFP(+)/ SSEA3(-)
**Gene**	R1	R2	R1	R2	R1	R2		
MIXL1	3	3	96	111	68	54	5.05	4.28

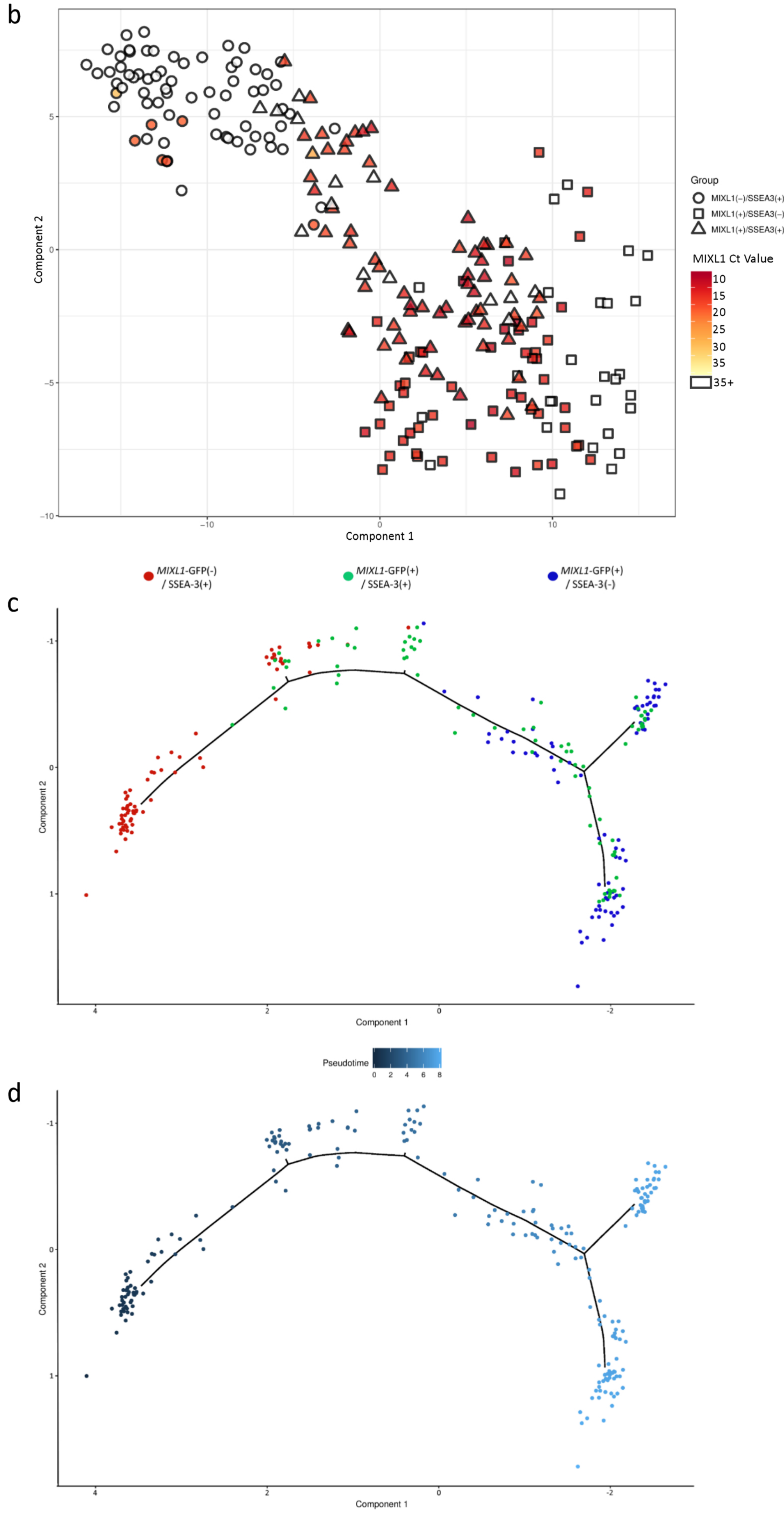

Supplementary Figure Legends

Supplementary figure 1: *MIXL1* Gene expression analysis and Pseudo-time projection.

a) Table of Fragments Per Kilobase per Million reads (FPKM) values for *MIXL1* from populations sorted for *MIXL1*-GFP and SSEA-3 (n=2 biological replicates) the LOG2 Fold change is also displayed comparing the other populations to the *MIXL1*-GFP(-)/SSEA-3(+) population. **b)** t-SNE plots of the expression of 45 genes (Supplementary Table 1) in individual cells measured by Fluidigm Biomark qPCR. All fractions are displayed in the same dimensional space: *MIXL1*-GFP(-)/SSEA-3(+) cells as circles, *MIXL1*-GFP(+)/SSEA-3(+) cells as squares and *MIXL1*-GFP(-)/SSEA-3(+) cells as triangles. Shape interiors are coloured by *MIXL1* Ct values. **c-d)** Minimal spanning tree trajectory. Each dot represents and individual cells expression profile, plotted in a two-dimensional independent component space according to Monocle2s pseudotime ordering. The solid black line represents the path of the trajectory. **c)** Cells are coloured according to the fraction which they are derived from *MIXL1*-GFP(-)/SSEA-3(+) (Red), *MIXL1*-GFP(+)/SSEA-3(+) (Green) and *MIXL1*-GFP(+)/SSEA-3(-) (Blue). **d)** Cells are coloured by their position in pseudotime. Note that the start of pseudotime and end of pseudotime match with the *MIXL1*-GFP(-)/SSEA-3(+) and *MIXL1*-GFP(+)/SSEA-3(-), respectively, with *MIXL1*-GFP(+)/SSEA-3(+) spanning the pseudotime space between. **b-d)** Source data are provided as a Source Data file.

SFig2

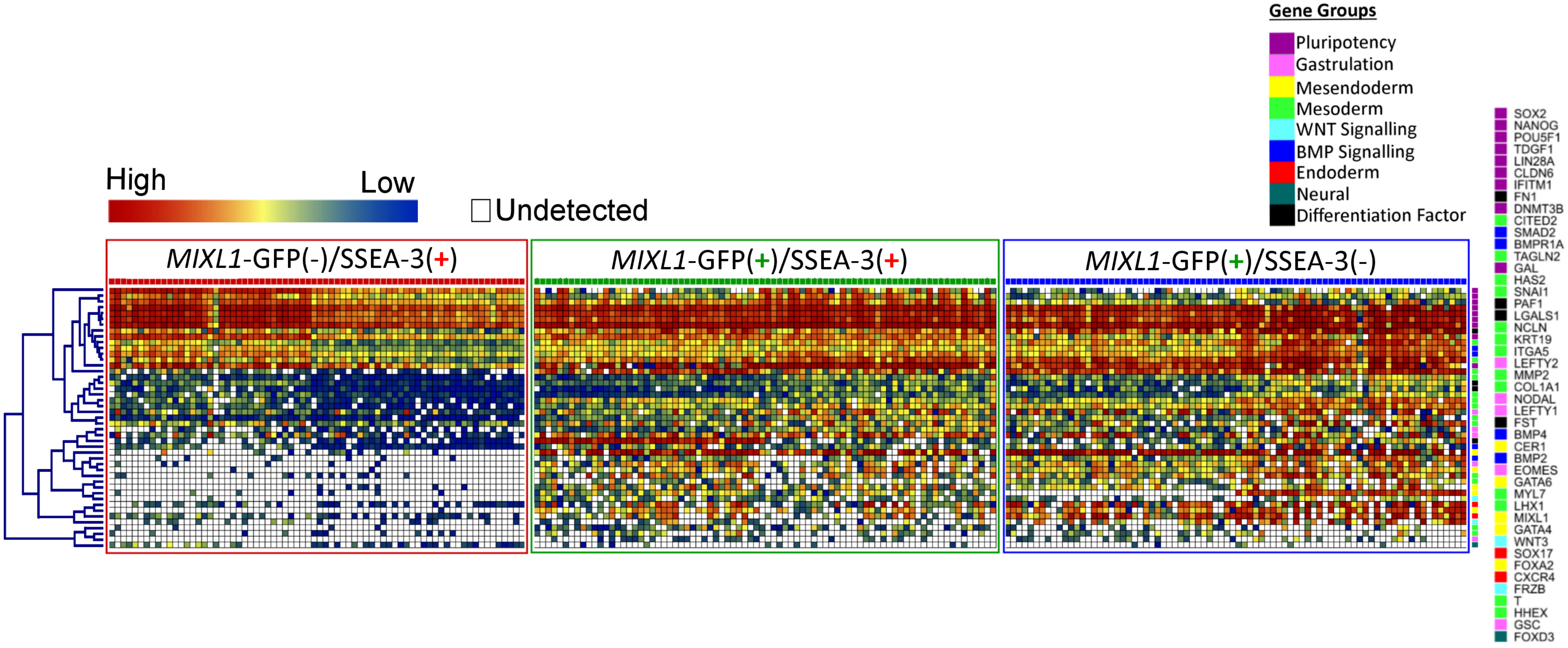
High
Low
Undetected
Gene Groups
Pluripotency
Gastrulation
Mesendoderm
Mesoderm
WNT Signalling
BMP Signalling
Endoderm
Neural
Differentiation Factor
MIXL1-GFP(-)/SSEA-3(+)
MIXL1-GFP(+)/SSEA-3(+)
MIXL1-GFP(+)/SSEA-3(-)
SOX2
NANOG
POU5F1
TDGF1
LIN28A
CLDN6
IFITM1
FN1
DNMT3B
CITED2
SMAD2
BMPR1A
TAGLN2
GAL
HAS2
SNAI1
PAF1
LGALS1
NCLN
KRT19
ITGA5
LEFTY2
MMP2
COL1A1
NODAL
LEFTY1
FST
BMP4
CER1
BMP2
EOMES
GATA6
MYL7
LHX1
MIXL1
GATA4
WNT3
SOX17
FOXA2
CXCR4
FRZB
T
HHEX
GSC
FOXD3

Supplementary figure 2: Single Cell qPCR analysis.

Heatmap analysis of 232 single cells analysed across 45 genes using a Fluidigm BioMark system, hierarchical clustering was performed for the genes assessed. The heatmap visualises the individual gene expression after normalisation across genes and samples. White coloured genes indicates undetected levels. Cells co-expressing pluripotency and differentiation associated genes were readily detected in the *MIXL1*-GFP(+)/SSEA-3(+) fraction but not the other fractions. Source data are provided as a Source Data file.

SFig3

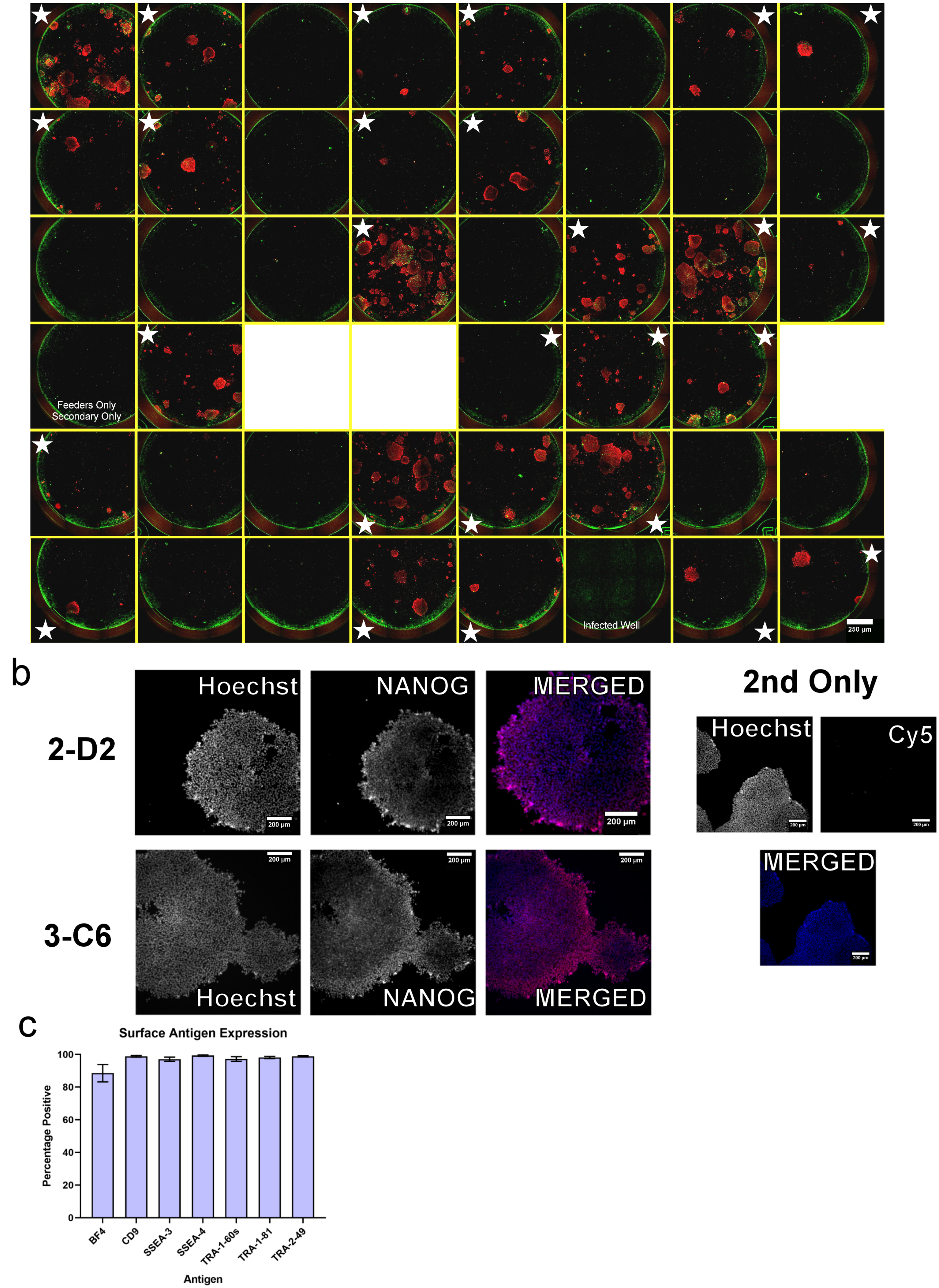
a
Feeders Only
Secondary Only
Infected Well
250 µm
b
2-D2
3-C6
Hoechst
NANOG
MERGED
200 µm
2nd Only
Cy5
c
Surface Antigen Expression
Percentage Positive
Antigen
BF4
CD9
SSEA-3
SSEA-4
TRA-1-60s
TRA-1-81
TRA-2-49

Supplementary figure 3: Assessing the stem cell potential of *MIXL1*-GFP(+)/SSEA-3(+) substate.

a) Live TRA-1-81 staining fluorescent images of colonies derived after the first passage into a 48 well plate, TRA-1-81(RED) and *MIXL1*-GFP (GREEN). Wells marked with white stars indicates clones that survived the passage and stained positive for TRA-1-81. Of the 44 colonies passaged, 27 survived and stained positive for TRA-1-81 (Scale Bar = 250µM). **b)** Representative immunofluorescent analysis of NANOG expression in HES3 *MIXL1*-GFP clones 2-D2 and 3-C6 growing in E8 conditions (all six clones assessed were positive for NANOG). Merged images display Hoechst (Nuclei) in blue and NANOG positive cells in magenta. Secondary only staining control is also shown (Scale Bar = 200µM). **c)** Bar chart showing the percentage positive cells from flow cytometry analysis for the stem cell associated antigens BF4, CD9, SSEA-3, SSEA4, TRA-1-60s, TRA-1-81 and TRA-2-49 for six clonal lines established (n= 6 independent clonal lines, Mean of all lines +/- SD). All lines displayed high expression of these surface markers. Source data are provided as a Source Data file.

SFig 4

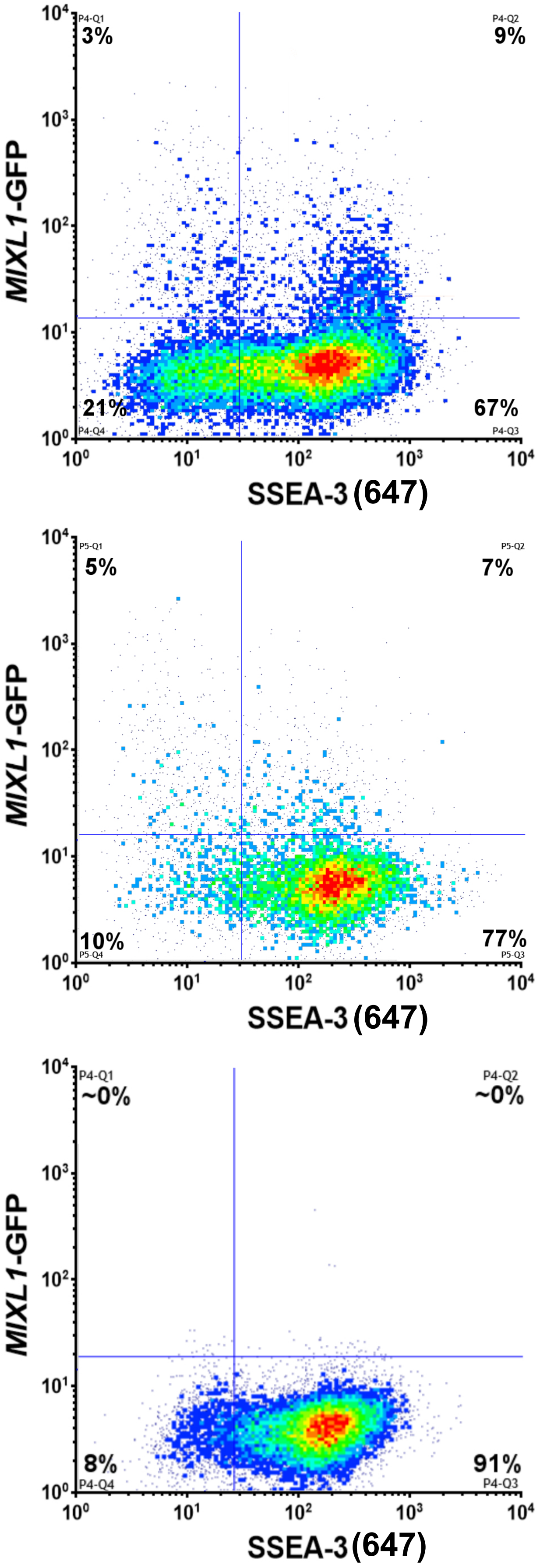

P4-Q1
3%
P4-Q2
9%
21%
P4-Q4
67%
P4-Q3
MIXL1-GFP
SSEA-3 (647)
P5-Q1
5%
P5-Q2
7%
10%
P5-Q4
77%
P5-Q3
MIXL1-GFP
SSEA-3 (647)
P4-Q1
~0%
P4-Q2
~0%
8%
P4-Q4
91%
P4-Q3
MIXL1-GFP
SSEA-3 (647)

Supplementary figure 4: *MIXL1*-GFP / SSEA-3 Co-expression in MEF/KOSR Conditions

Flow cytometry density plots of *MIXL1*-GFP versus SSEA-3 from cells grown in MEF/KOSR conditions, showing variable expression (3 independent cell cultures are displayed).

SFig 5

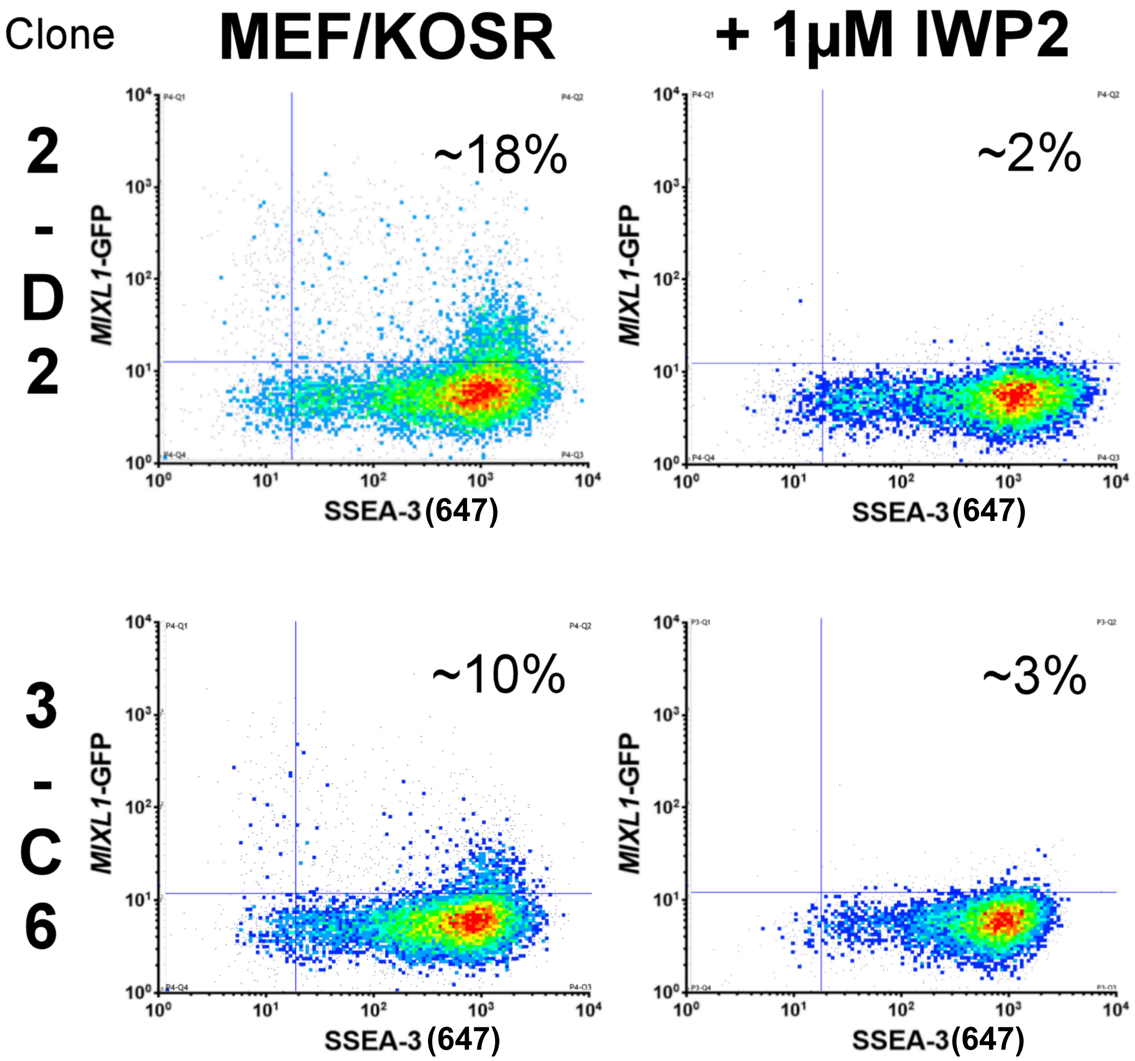

Supplementary figure 5: The addition of IWP-2 into MEF/KOSR conditions decreases the proportion of MIXL1(+)/SSEA-3(+) cells.

Flow cytometry density plots of clonal lines 2-D2 and 3-C6 under MEF/KOSR conditions with and without the addition of 1µM IWP-2. X axis displays SSEA-3 expression level, Y Axis *MIXL1*-GFP expression. The percentage of *MIXL1*-GFP(+)/SSEA-3(+) is shown on each density plot.

Sfig 6

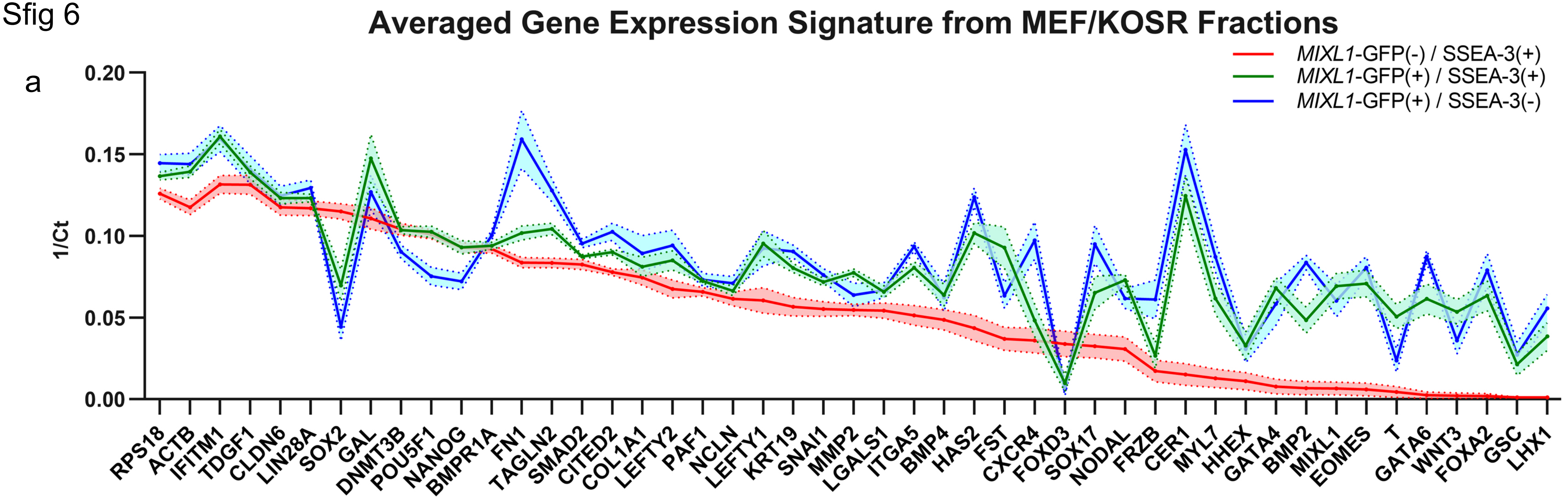
a
Averaged Gene Expression Signature from MEF/KOSR Fractions
MIXL1-GFP(-) / SSEA-3(+)
MIXL1-GFP(+) / SSEA-3(+)
MIXL1-GFP(+) / SSEA-3(-)
1/Ct
0.20
0.15
0.10
0.05
0.00
RPS18
ACTB
IFITM1
TDGF1
CLDN6
LIN28A
SOX2
GAL
DNMT3B
POU5F1
NANOG
BMPR1A
FN1
TAGLN2
SMAD2
CITED2
COL1A1
LEFTY2
PAF1
NCLN
LEFTY1
KRT19
SNAI1
MMP2
LGALS1
ITGA5
BMP4
HAS2
FST
CXCR4
FOXD3
SOX17
NODAL
FRZB
CER1
MYL7
HHEX
GATA4
BMP2
MIXL1
EOMES
T
GATA6
WNT3
FOXA2
GSC
LHX1

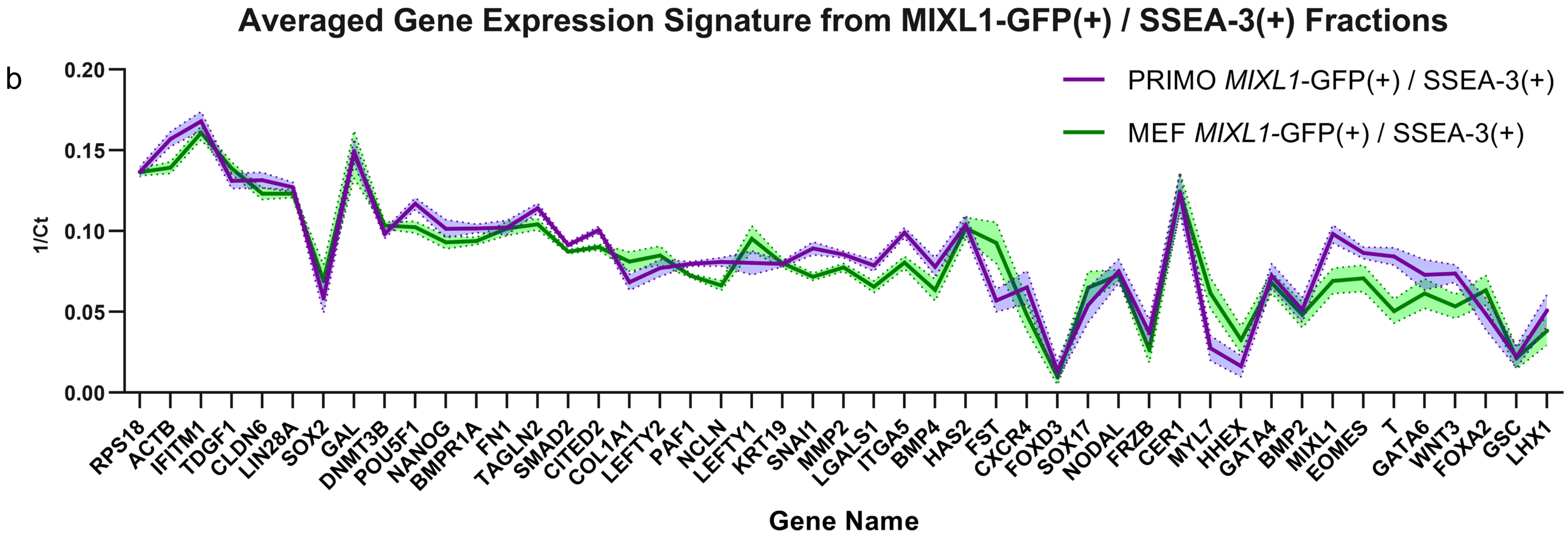
b
Averaged Gene Expression Signature from MIXL1-GFP(+) / SSEA-3(+) Fractions
PRIMO MIXL1-GFP(+) / SSEA-3(+)
MEF MIXL1-GFP(+) / SSEA-3(+)
1/Ct
0.20
0.15
0.10
0.05
0.00
RPS18
ACTB
IFITM1
TDGF1
CLDN6
LIN28A
SOX2
GAL
DNMT3B
POU5F1
NANOG
BMPR1A
FN1
TAGLN2
SMAD2
CITED2
COL1A1
LEFTY2
PAF1
NCLN
LEFTY1
KRT19
SNAI1
MMP2
LGALS1
ITGA5
BMP4
HAS2
FST
CXCR4
FOXD3
SOX17
NODAL
FRZB
CER1
MYL7
HHEX
GATA4
BMP2
MIXL1
EOMES
T
GATA6
WNT3
FOXA2
GSC
LHX1
Gene Name

Supplementary figure 6: Averaged qPCR Signature Comparison

The average 1/Ct values for 47 genes from single cell qPCR analysis. Genes were ordered from highest to lowest expression based on the *MIXL1*-GFP(-)/SSEA-3(+) fraction. A solid line connects the mean expression points to give a state "signature" with surrounding shaded area represents the 95% confidence interval of the data. **a)** Displays the state signatures of *MIXL1*-GFP(-)/SSEA-3(+)(red), *MIXL1*-GFP(+)/SSEA-3(+) (green) and *MIXL1*-GFP(+)/SSEA-3(-) (blue) grown in MEF/KOSR conditions. **b)** Displays the state signatures of *MIXL1*-GFP(+)/SSEA-3(+) cells grown in MEF/KOSR (green) and Primo (purple) conditions. The state signature of the both *MIXL1*-GFP(+)/SSEA-3(+) were very similar. Source data are provided as a Source Data file.

SFig 7a

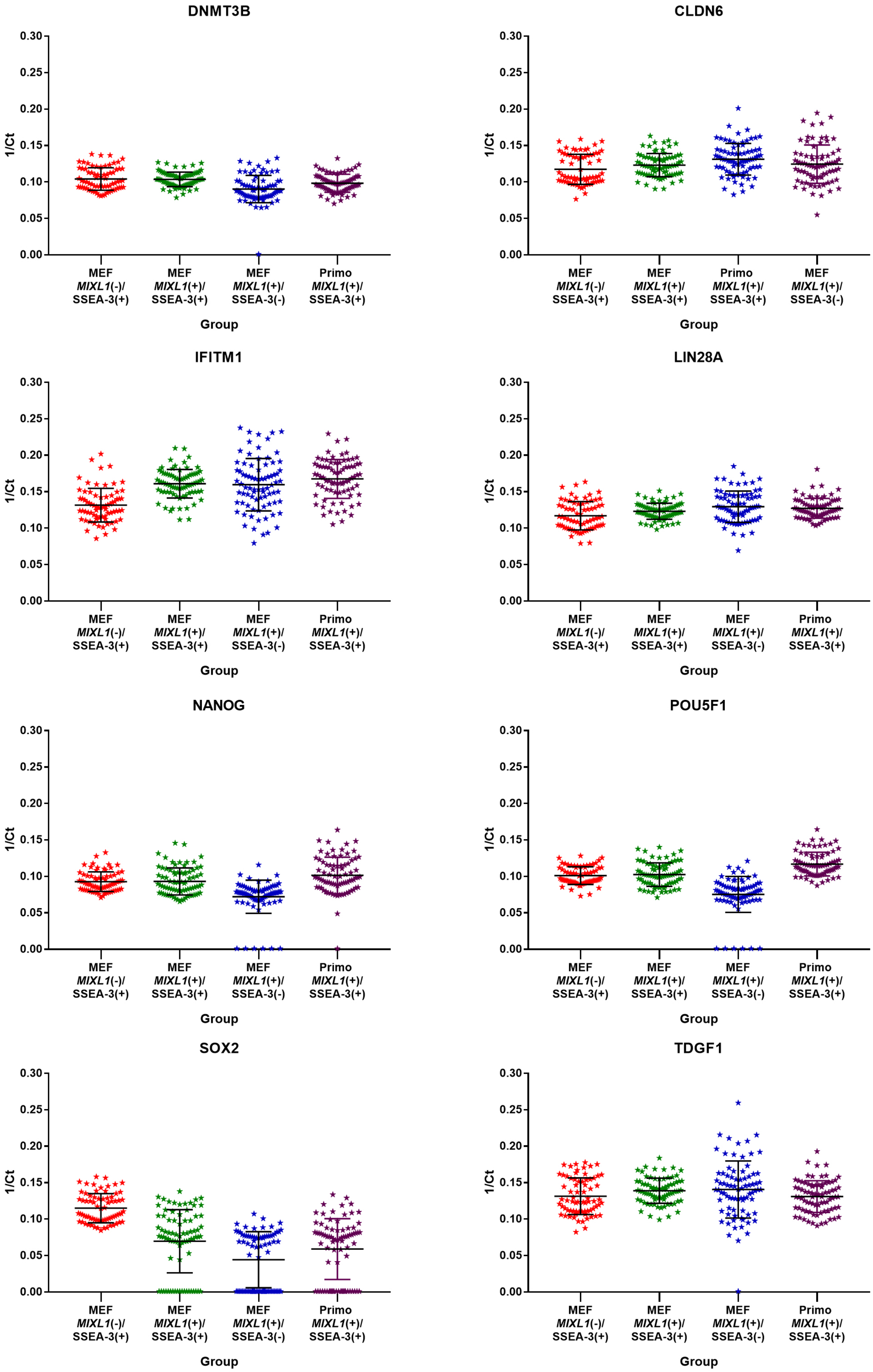
DNMT3B
1/Ct
0.30
0.25
0.20
0.15
0.10
0.05
0.00
MEF *MIXL1*(-)/ SSEA-3(+)
MEF *MIXL1*(+)/ SSEA-3(+)
MEF *MIXL1*(+)/ SSEA-3(-)
Primo *MIXL1*(+)/ SSEA-3(+)
Group
CLDN6
1/Ct
0.30
0.25
0.20
0.15
0.10
0.05
0.00
MEF *MIXL1*(-)/ SSEA-3(+)
MEF *MIXL1*(+)/ SSEA-3(+)
Primo *MIXL1*(+)/ SSEA-3(+)
MEF *MIXL1*(+)/ SSEA-3(-)
Group
IFITM1
1/Ct
0.30
0.25
0.20
0.15
0.10
0.05
0.00
MEF *MIXL1*(-)/ SSEA-3(+)
MEF *MIXL1*(+)/ SSEA-3(+)
MEF *MIXL1*(+)/ SSEA-3(-)
Primo *MIXL1*(+)/ SSEA-3(+)
Group
LIN28A
1/Ct
0.30
0.25
0.20
0.15
0.10
0.05
0.00
MEF *MIXL1*(-)/ SSEA-3(+)
MEF *MIXL1*(+)/ SSEA-3(+)
MEF *MIXL1*(+)/ SSEA-3(-)
Primo *MIXL1*(+)/ SSEA-3(+)
Group
NANOG
1/Ct
0.30
0.25
0.20
0.15
0.10
0.05
0.00
MEF *MIXL1*(-)/ SSEA-3(+)
MEF *MIXL1*(+)/ SSEA-3(+)
MEF *MIXL1*(+)/ SSEA-3(-)
Primo *MIXL1*(+)/ SSEA-3(+)
Group
POU5F1
1/Ct
0.30
0.25
0.20
0.15
0.10
0.05
0.00
MEF *MIXL1*(-)/ SSEA-3(+)
MEF *MIXL1*(+)/ SSEA-3(+)
MEF *MIXL1*(+)/ SSEA-3(-)
Primo *MIXL1*(+)/ SSEA-3(+)
Group
SOX2
1/Ct
0.30
0.25
0.20
0.15
0.10
0.05
0.00
MEF *MIXL1*(-)/ SSEA-3(+)
MEF *MIXL1*(+)/ SSEA-3(+)
MEF *MIXL1*(+)/ SSEA-3(-)
Primo *MIXL1*(+)/ SSEA-3(+)
Group
TDGF1
1/Ct
0.30
0.25
0.20
0.15
0.10
0.05
0.00
MEF *MIXL1*(-)/ SSEA-3(+)
MEF *MIXL1*(+)/ SSEA-3(+)
MEF *MIXL1*(+)/ SSEA-3(-)
Primo *MIXL1*(+)/ SSEA-3(+)
Group

SFig 7b

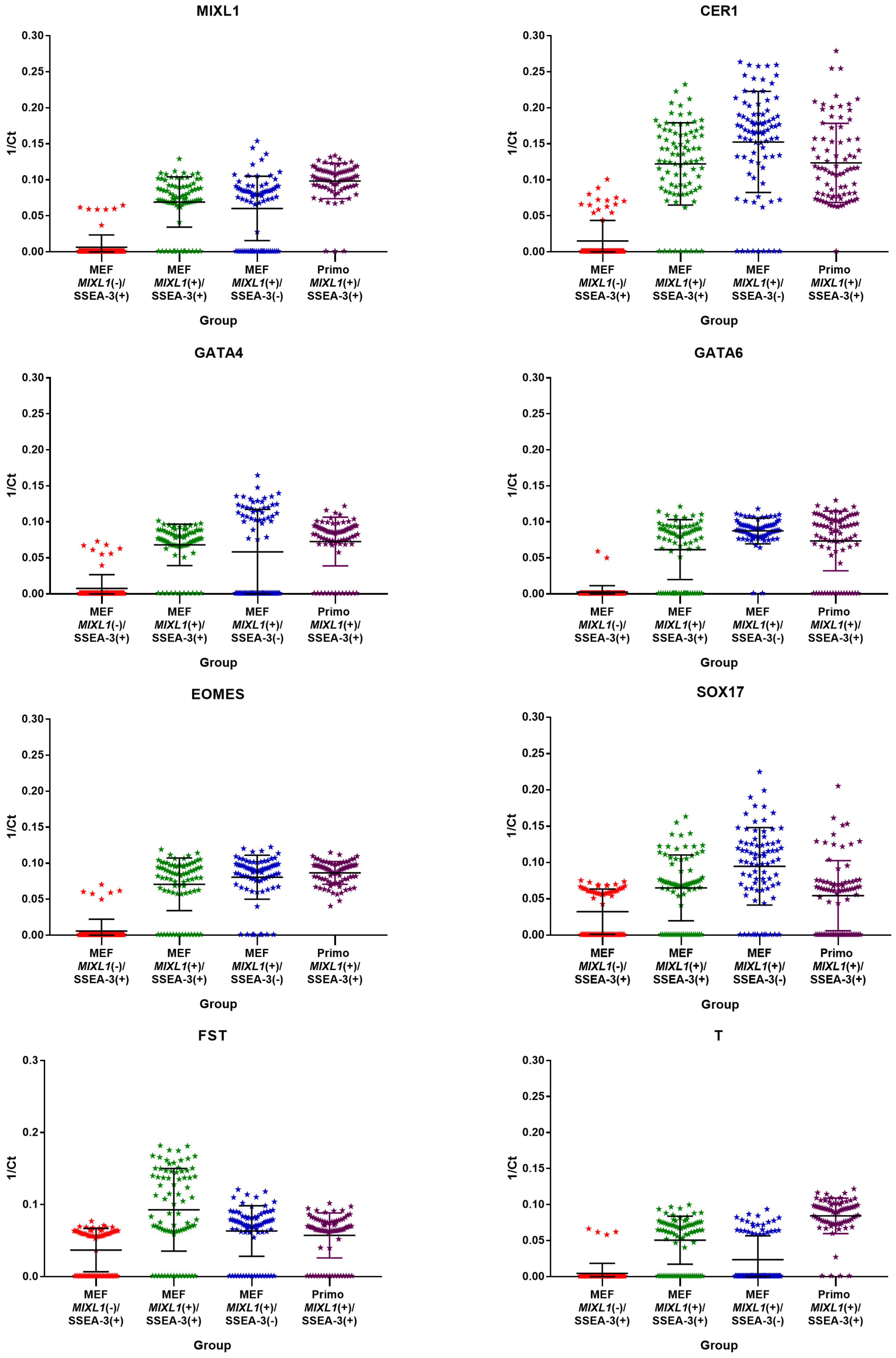
MIXL1
CER1
GATA4
GATA6
EOMES
SOX17
FST
T
1/Ct
Group
MEF
MIXL1(-)/
SSEA-3(+)
MEF
MIXL1(+)/
SSEA-3(+)
MEF
MIXL1(+)/
SSEA-3(-)
Primo
MIXL1(+)/
SSEA-3(+)

SFig 7c

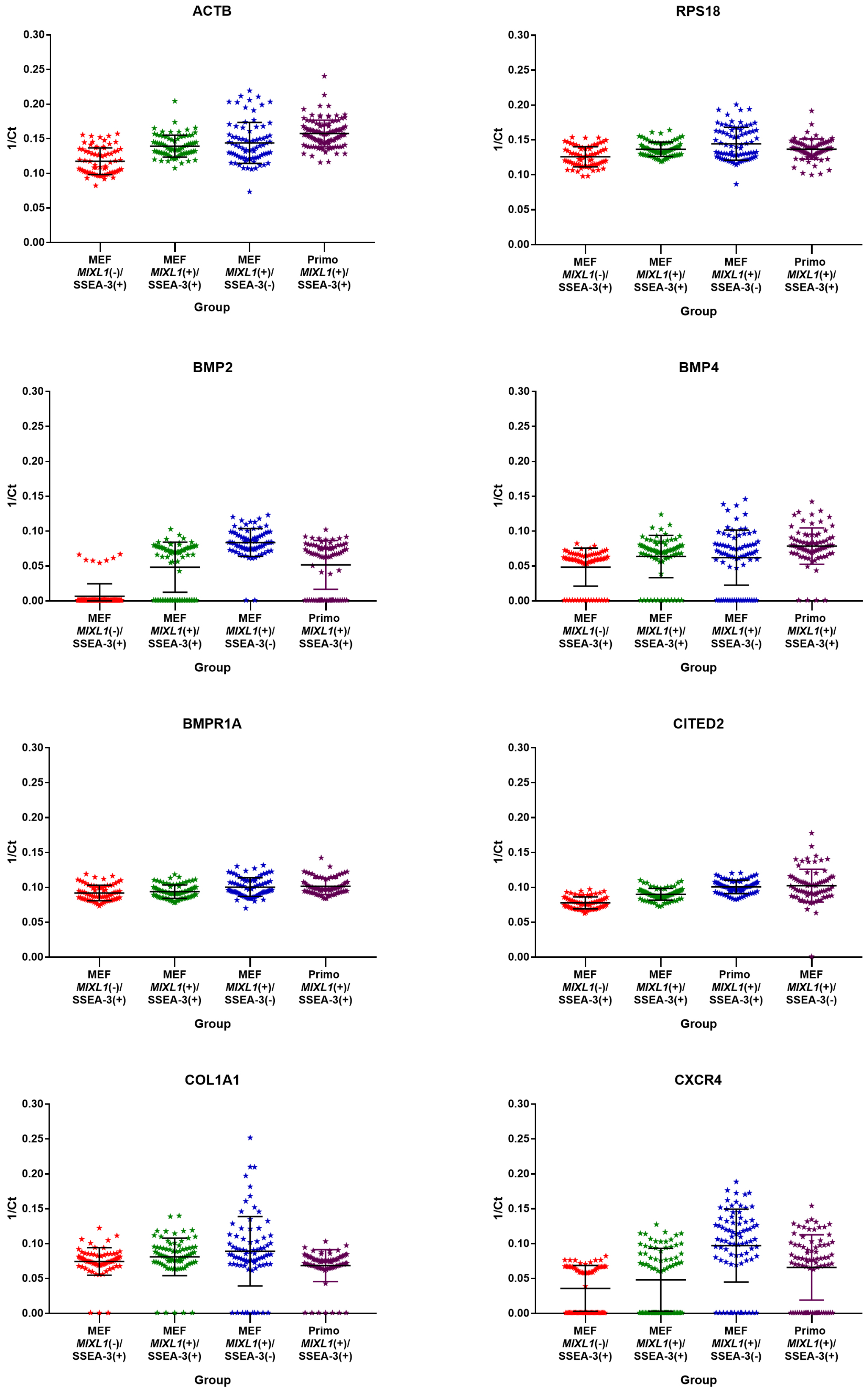
ACTB
RPS18
BMP2
BMP4
BMPR1A
CITED2
COL1A1
CXCR4
1/Ct
0.30
0.25
0.20
0.15
0.10
0.05
0.00
MEF MIXL1(-)/ SSEA-3(+)
MEF MIXL1(+)/ SSEA-3(+)
MEF MIXL1(+)/ SSEA-3(-)
Primo MIXL1(+)/ SSEA-3(+)
Group

SFig 7d

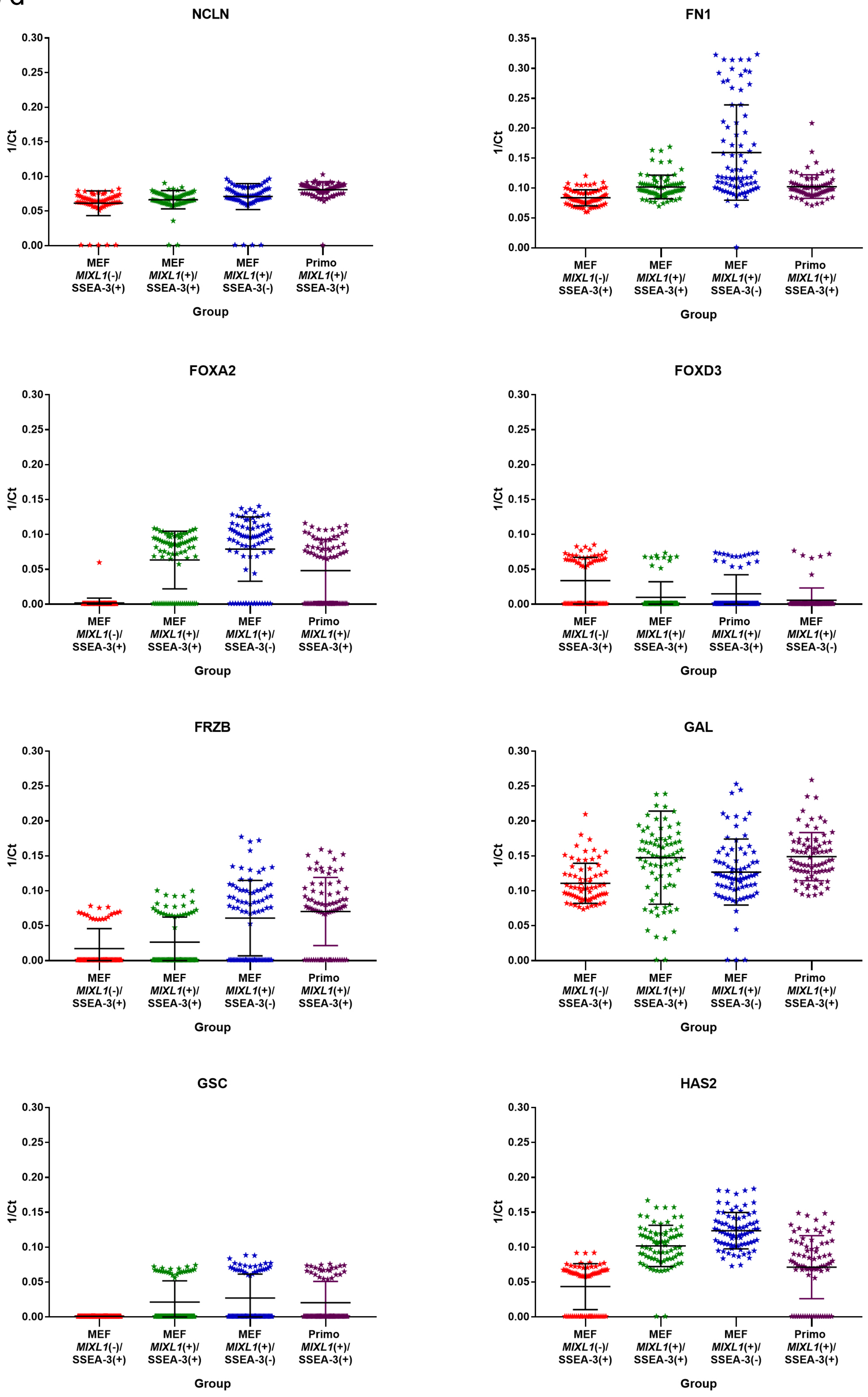
NCLN
1/Ct
0.30
0.25
0.20
0.15
0.10
0.05
0.00
MEF MIXL1(-)/ SSEA-3(+)
MEF MIXL1(+)/ SSEA-3(+)
MEF MIXL1(+)/ SSEA-3(-)
Primo MIXL1(+)/ SSEA-3(+)
Group
FN1
1/Ct
0.35
0.30
0.25
0.20
0.15
0.10
0.05
0.00
MEF MIXL1(-)/ SSEA-3(+)
MEF MIXL1(+)/ SSEA-3(+)
MEF MIXL1(+)/ SSEA-3(-)
Primo MIXL1(+)/ SSEA-3(+)
Group
FOXA2
1/Ct
0.30
0.25
0.20
0.15
0.10
0.05
0.00
MEF MIXL1(-)/ SSEA-3(+)
MEF MIXL1(+)/ SSEA-3(+)
MEF MIXL1(+)/ SSEA-3(-)
Primo MIXL1(+)/ SSEA-3(+)
Group
FOXD3
1/Ct
0.30
0.25
0.20
0.15
0.10
0.05
0.00
MEF MIXL1(-)/ SSEA-3(+)
MEF MIXL1(+)/ SSEA-3(+)
Primo MIXL1(+)/ SSEA-3(+)
MEF MIXL1(+)/ SSEA-3(-)
Group
FRZB
1/Ct
0.30
0.25
0.20
0.15
0.10
0.05
0.00
MEF MIXL1(-)/ SSEA-3(+)
MEF MIXL1(+)/ SSEA-3(+)
MEF MIXL1(+)/ SSEA-3(-)
Primo MIXL1(+)/ SSEA-3(+)
Group
GAL
1/Ct
0.30
0.25
0.20
0.15
0.10
0.05
0.00
MEF MIXL1(-)/ SSEA-3(+)
MEF MIXL1(+)/ SSEA-3(+)
MEF MIXL1(+)/ SSEA-3(-)
Primo MIXL1(+)/ SSEA-3(+)
Group
GSC
1/Ct
0.30
0.25
0.20
0.15
0.10
0.05
0.00
MEF MIXL1(-)/ SSEA-3(+)
MEF MIXL1(+)/ SSEA-3(+)
MEF MIXL1(+)/ SSEA-3(-)
Primo MIXL1(+)/ SSEA-3(+)
Group
HAS2
1/Ct
0.30
0.25
0.20
0.15
0.10
0.05
0.00
MEF MIXL1(-)/ SSEA-3(+)
MEF MIXL1(+)/ SSEA-3(+)
MEF MIXL1(+)/ SSEA-3(-)
Primo MIXL1(+)/ SSEA-3(+)
Group

SFig 7e

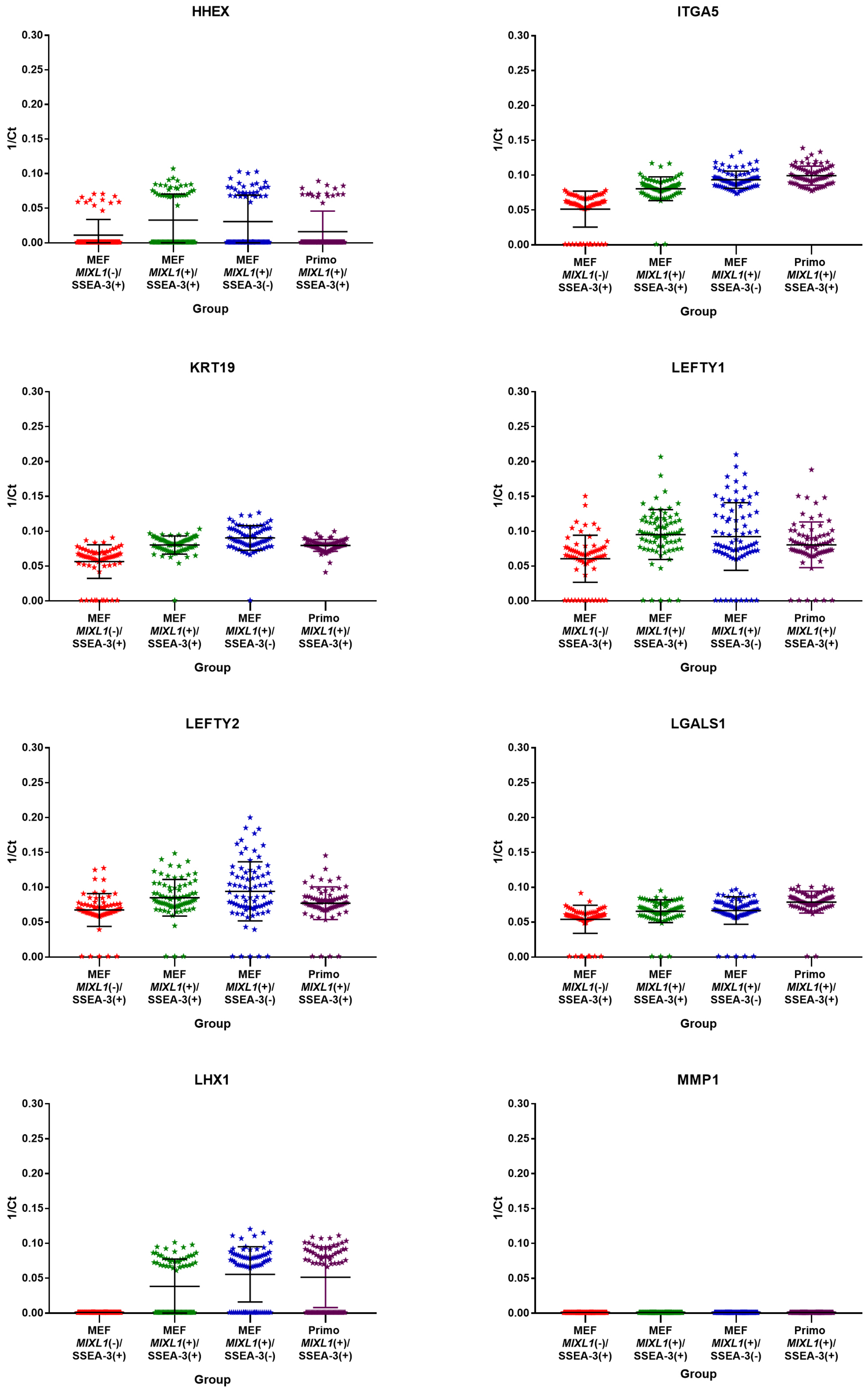
HHEX
ITGA5
KRT19
LEFTY1
LEFTY2
LGALS1
LHX1
MMP1
1/Ct
0.30
0.25
0.20
0.15
0.10
0.05
0.00
MEF MIXL1(-)/ SSEA-3(+)
MEF MIXL1(+)/ SSEA-3(+)
MEF MIXL1(+)/ SSEA-3(-)
Primo MIXL1(+)/ SSEA-3(+)
Group

SFig 7f

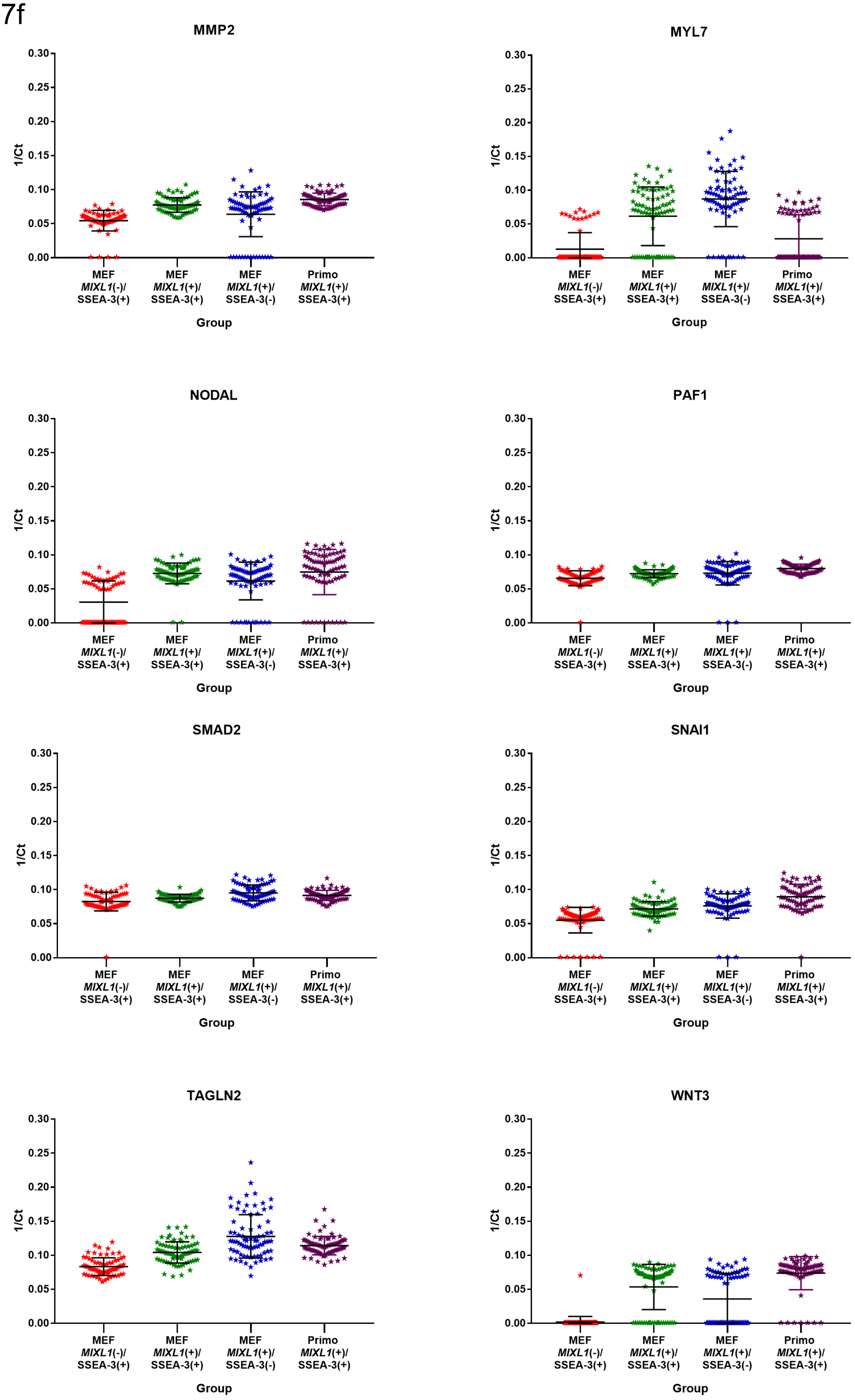
MMP2
MYL7
NODAL
PAF1
SMAD2
SNAI1
TAGLN2
WNT3
1/Ct
0.00
0.05
0.10
0.15
0.20
0.25
0.30
MEF MIXL1(-)/ SSEA-3(+)
MEF MIXL1(+)/ SSEA-3(+)
MEF MIXL1(+)/ SSEA-3(-)
Primo MIXL1(+)/ SSEA-3(+)
Group

Supplementary figure 7: Single Cell Gene Expression Plots.

The single cell gene expression distribution was similar between the two *MIXL1*-GFP(+)/SSEA-3(+) fractions from MEF/KOSR (green) and PRIMO (purple) conditions. 1/Ct values for each single cell for a given gene. Mean and standard deviation are displayed on top of data sets as black bars. Cells are split into their respective sorted fractions MEF/KOSR conditions *MIXL1*-GFP(-)/SSEA-3(+) cells in red, *MIXL1*-GFP(+)/SSEA-3(+) cells in green, *MIXL1*-GFP(-)/SSEA-3(+) cells in blue and PRIMO conditions *MIXL1*-GFP(+)/SSEA-3(+) cells in purple. **a)** Contains a collection of plots from genes associated with pluripotency. **b)** Contains a collection of plots from key genes associated with mesendoderm differentiation. **c-f)** Contains plots from the remaining genes assessed by single cell qPCR. (n = 72 single cells for *MIXL1*-GFP(-)/SSEA-3(+) from MEF/KOSR and n = 80 single cells for all other fractions). Source data are provided as a Source Data file.

SFig8

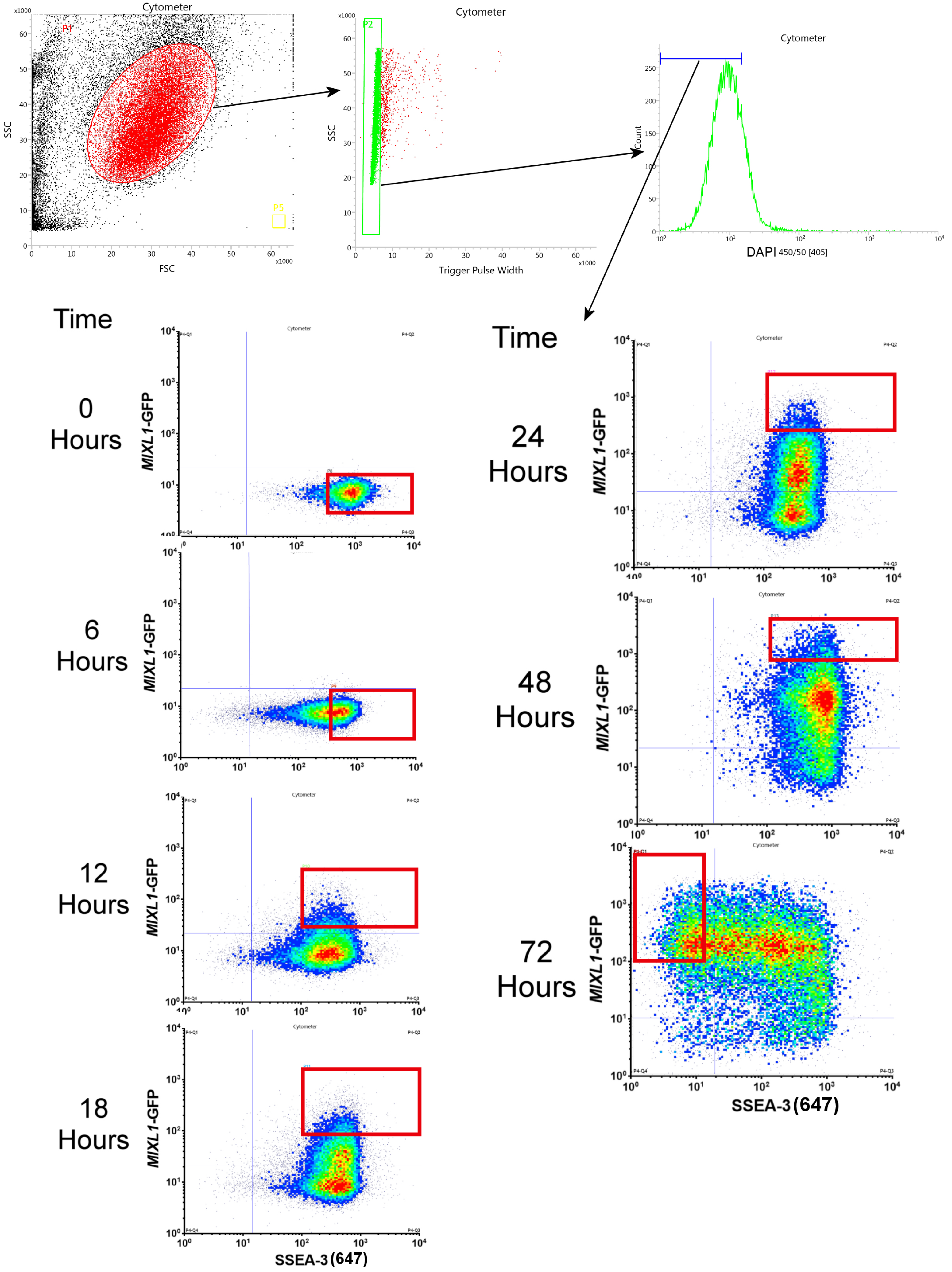

Cytometer
SSC
FSC
P1
P5
P2
Trigger Pulse Width
Count
DAPI 450/50 [405]
Time
0 Hours
6 Hours
12 Hours
18 Hours
24 Hours
48 Hours
72 Hours
MIXL1-GFP
SSEA-3(647)

Supplementary figure 8: Differentiation Time Course

Gating strategy for fluorescence activated cell sorting for the emerging fraction at each time point. A scatter plot of Forward Scatter (FSC) versus Side scatter (SSC) is used to set the P1 gate in red, which identifies the cell population. Doublet discrimination is performed using Trigger Pulse width versus SSC, P2 in green identifies single cells. A histogram plot was used for Live/Dead discrimination. DAPI staining is analysed with excitation by the violet laser 405nm and emission filter 450/50. P3 in blue identifies live cells. Live cells were then assessed on density plots of HES3 *MIXL1*-GFP cells stained for SSEA-3 at indicated time points after induction of differentiation in E8 containing 3μM CHIRON. Red boxes indicate the sorting gates for each timepoint. The expression of *MIXL1*-GFP increases first, before the eventual loss of SSEA-3.

SFig9

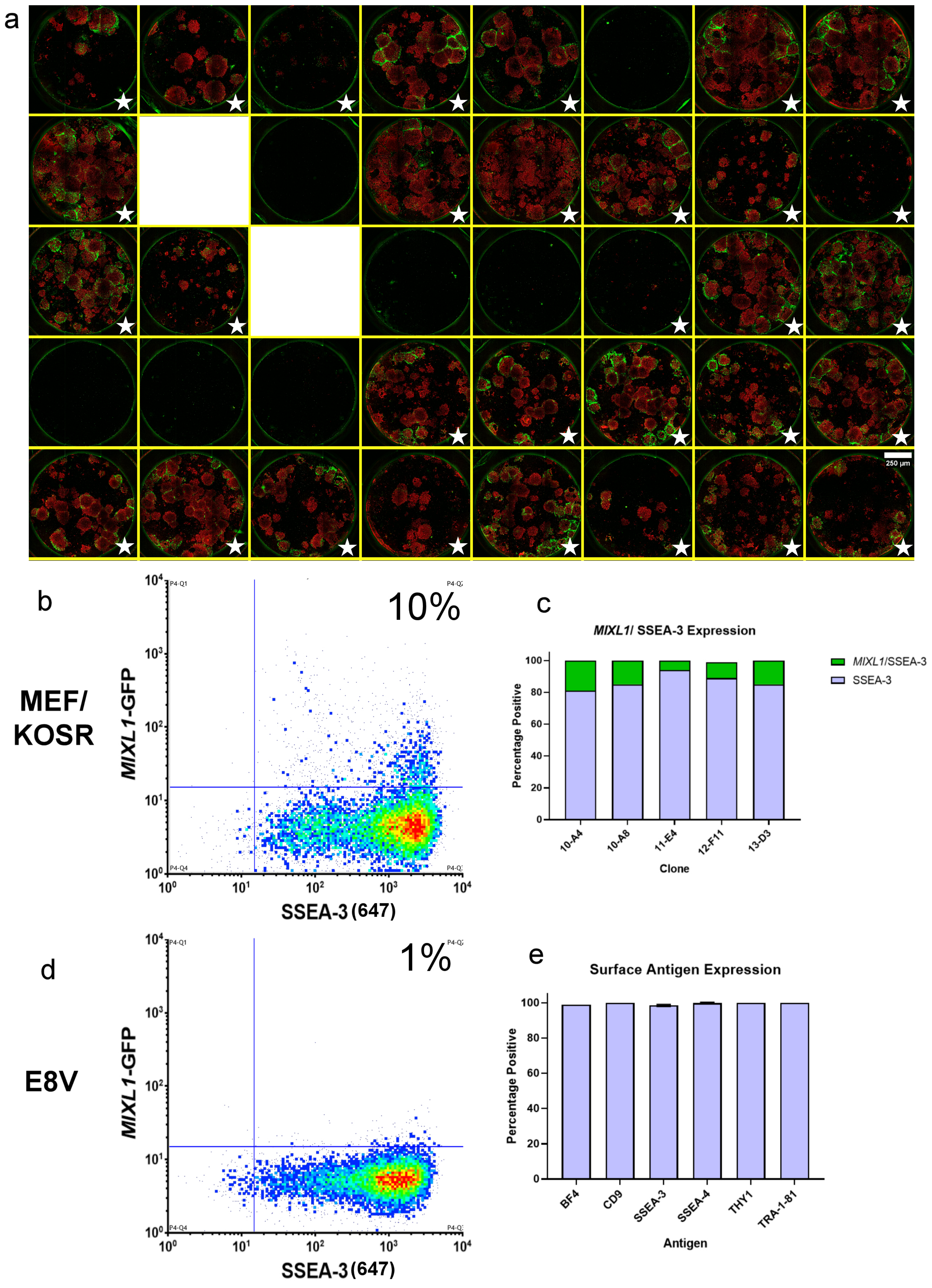
a
250 µm
b
MEF/
KOSR
10%
MIXL1-GFP
SSEA-3 (647)
c
MIXL1/ SSEA-3 Expression
MIXL1/SSEA-3
SSEA-3
Percentage Positive
10-A4
10-A8
11-E4
12-F11
13-D3
Clone
d
E8V
1%
MIXL1-GFP
SSEA-3 (647)
e
Surface Antigen Expression
Percentage Positive
BF4
CD9
SSEA-3
SSEA-4
THY1
TRA-1-81
Antigen

Supplementary figure 9: Clones generated from the *MIXL1*-GFP(+)/SSEA-3(+) from PRIMO medium exhibit normal stem cell growth and characteristics.

a) Live TRA-1-81 staining fluorescent images of colonies derived from single cell deposition of *MIXL1*-GFP(+)/SSEA-3(+) from PRIMO Plus conditions after the first passage into a 48 well plate, TRA-1-81(RED) and *MIXL1*-GFP (GREEN). Wells marked with white stars indicates clones that survived the passage and stained positive for TRA-1-81, 31 out of 38 passaged (Scale Bar = 250µM). **b)** Flow cytometry density plot of *MIXL1*-GFP versus SSEA-3 from clone 12-F11 grown in MEF/KOSR conditions. **c)** Bar chart of percentage positive stem cell associated antigen SSEA-3 and *MIXL1*-GFP expression for five clonal lines during initial expansion in MEF/KOSR conditions (n = 1 independent clone analysed at passage 3). **d)** Flow cytometry density plot of *MIXL1*-GFP versus SSEA-3 from clone 12-F11 after being transitioned into E8 conditions. **e)** Bar chart of percentage positive stem cell associated antigens BF4, CD9, SSEA-3, SSEA4, THY-1 and TRA-1-81 for six clonal lines (n= 6 independent clonal lines, Mean of all lines +/- SD). All lines displayed high expression of these surface markers. **c) e)** Source data are provided as a Source Data file.

SFig 10

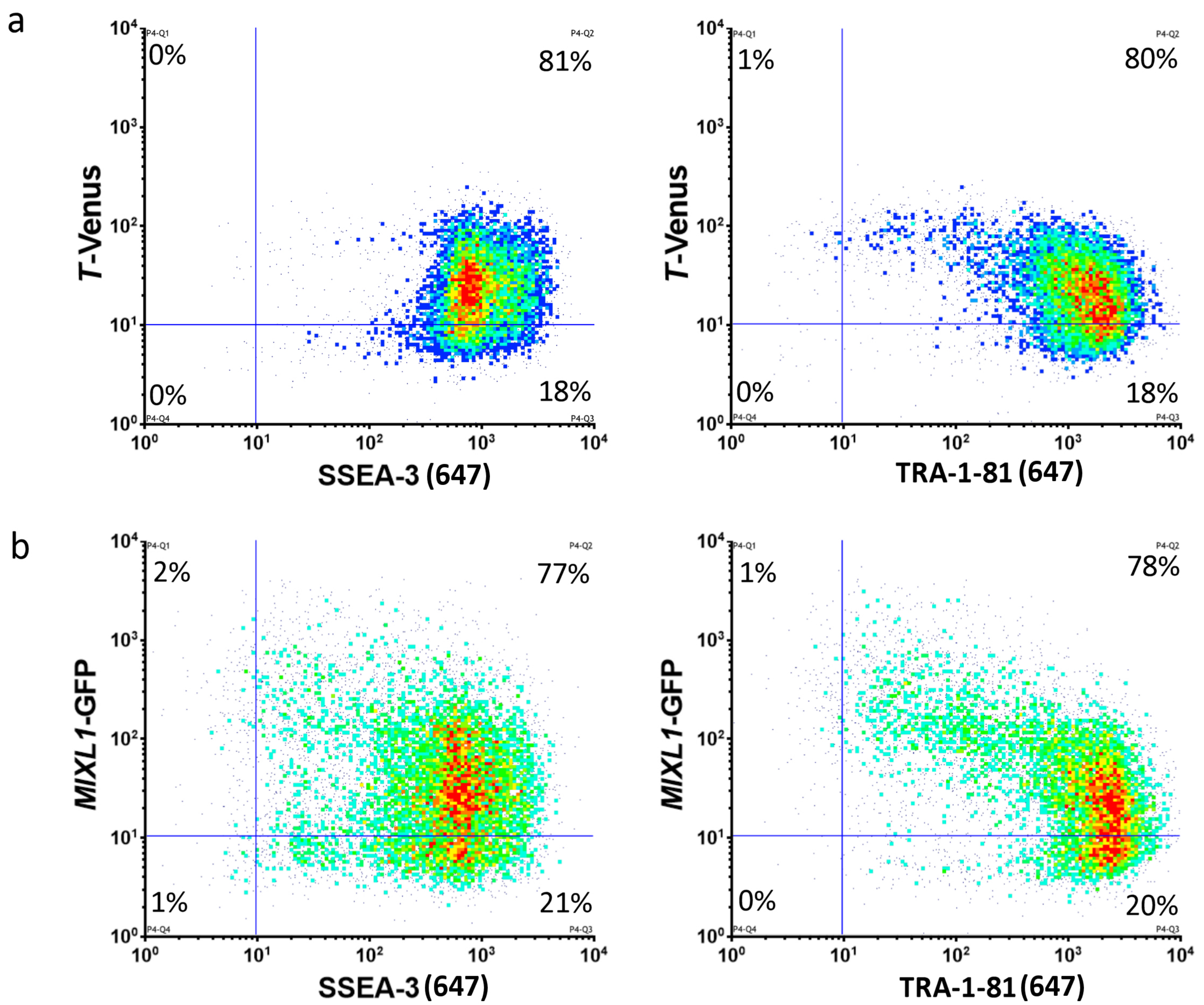
a
T-Venus
SSEA-3 (647)
0%
81%
0%
18%
T-Venus
TRA-1-81 (647)
1%
80%
0%
18%
b
MIXL1-GFP
SSEA-3 (647)
2%
77%
1%
21%
MIXL1-GFP
TRA-1-81 (647)
1%
78%
0%
20%

Supplementary figure 10: Pluripotency associated markers and reporter gene expression in PRIMO cultures.

Cells were analysed for pluripotency associated markers and reporter gene expression after three days in PRIMO Plus prior to the blood differentiation (Fig 5e). **a)** Flow cytometry density plots of *T*-Venus versus SSEA-3 or TRA-1-81. **b)** Flow cytometry density plots of *MIXL1*-GFP versus SSEA-3 or TRA-1-81.

SFig 11

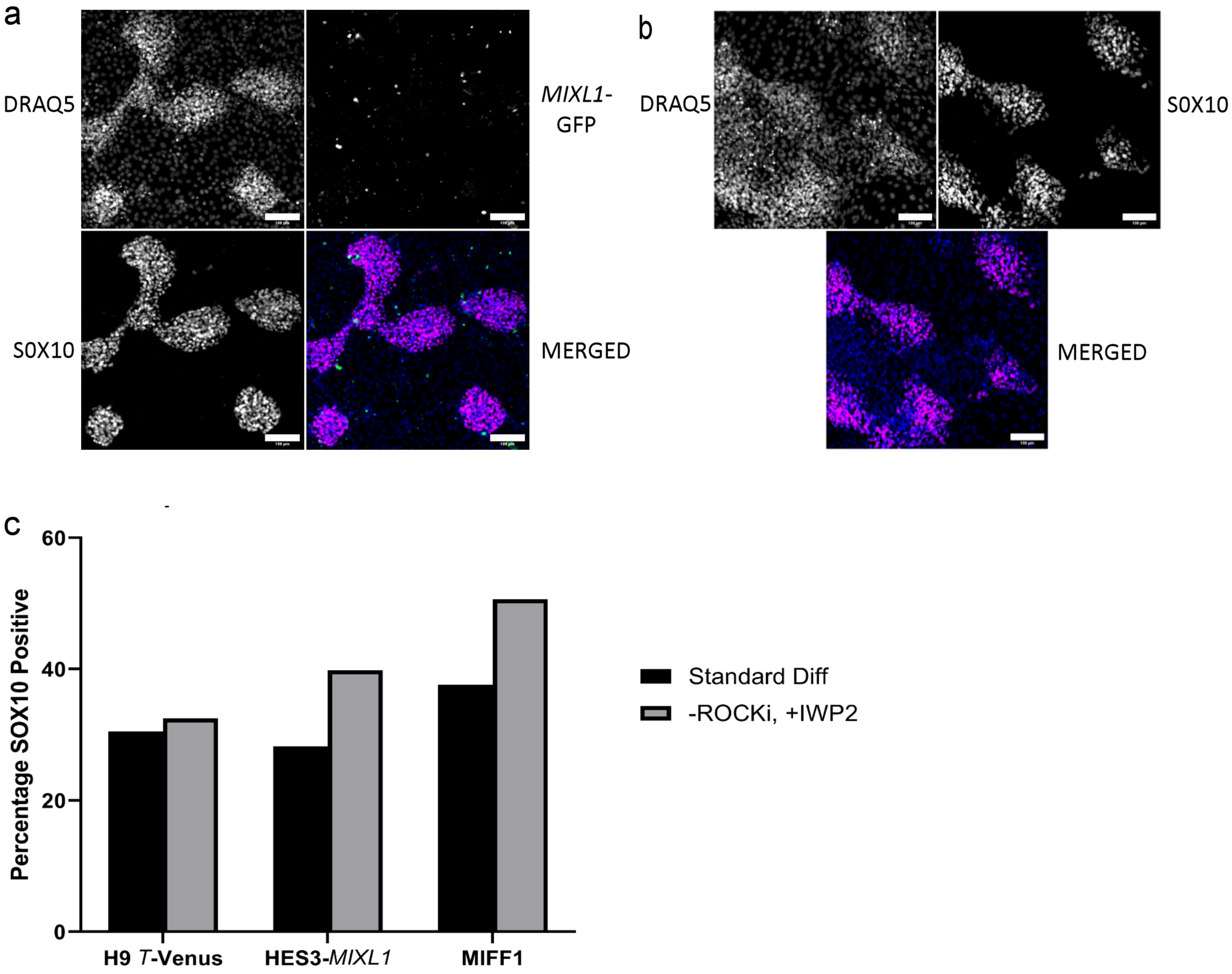
a
DRAQ5
MIXL1-
GFP
S0X10
MERGED
b
DRAQ5
S0X10
MERGED
c
Percentage SOX10 Positive
60
40
20
0
H9 T-Venus
HES3-MIXL1
MIFF1
Standard Diff
-ROCKi, +IWP2

Supplementary figure 11: Cells grown in PRIMO can generate SOX10 positive cells under neural crest differentiation.

a-b) Representative immunofluorescent images of SOX10 expression at day 5 of Neural crest differentiation of **a)** H9 *T*-Venus or **b)** MIFF1 from PRIMO Plus cultures. A merged image is present showing **a-b)** DRAQ5 (Blue) and SOX10 (Magenta) and **a)** *T*-Venus (Green) (Scale bar = 100μm). **c)** Percentage of SOX10 positive cells at day 5 of Neural crest differentiation for H9 *T*-Venus, HES3-*MIXL1* and MIFF1 under standard differentiation or without ROCK inhibitor (ROCKi) and 1μM IWP-2 for the first two days of the differentiation. (n = ~100,000 cells of 1 biological replicate from 3 separate cell lines, bars indicate percentage positive). Source data are provided as a Source Data file.

SFig 12

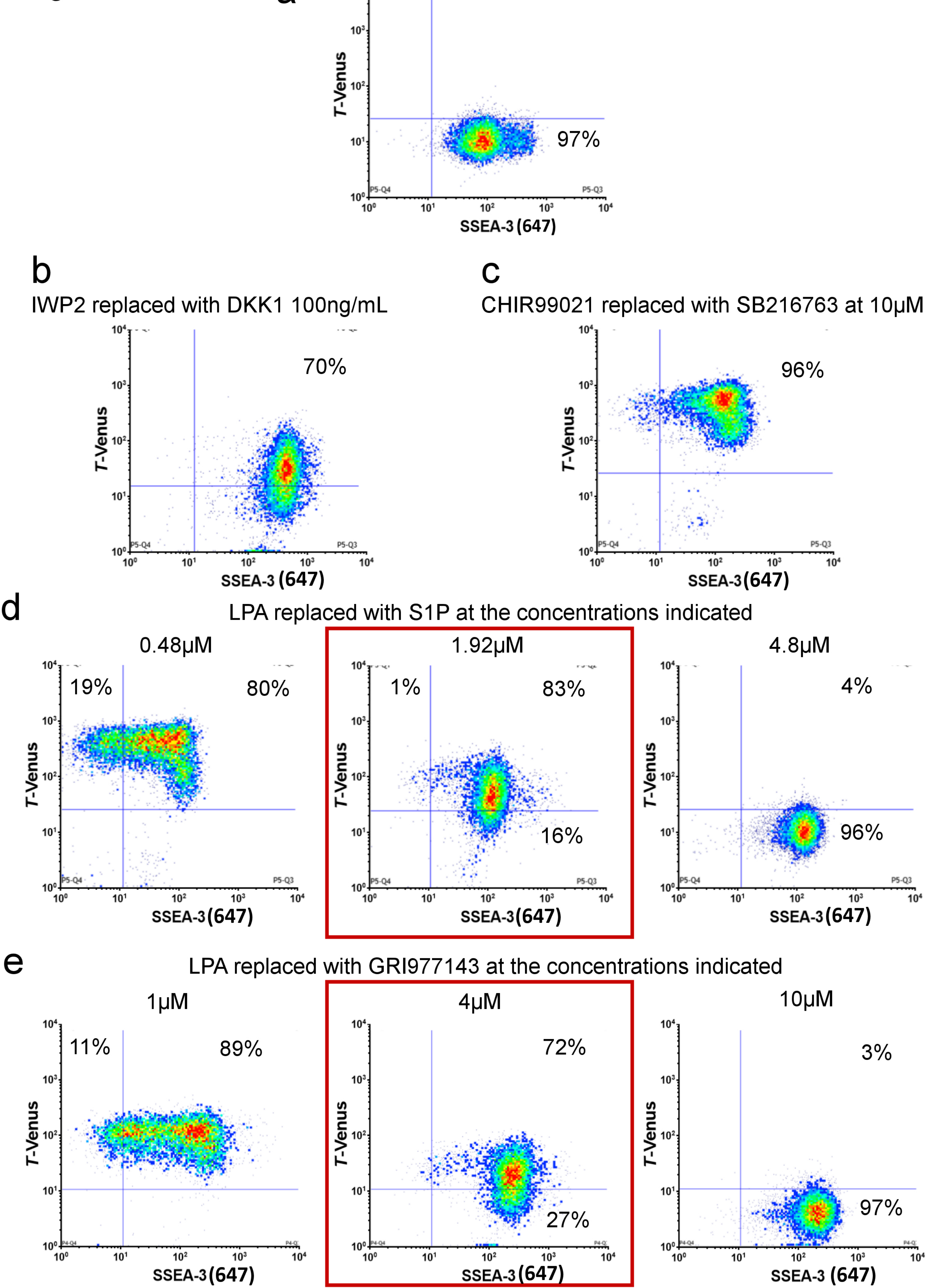
a
E8
T-Venus
SSEA-3 (647)
97%
b
IWP2 replaced with DKK1 100ng/mL
70%
c
CHIR99021 replaced with SB216763 at 10μM
96%
d
LPA replaced with S1P at the concentrations indicated
0.48μM
19%
80%
1.92μM
1%
83%
16%
4.8μM
4%
96%
e
LPA replaced with GRI977143 at the concentrations indicated
1μM
11%
89%
4μM
72%
27%
10μM
3%
97%

Supplementary figure 12: Components of PRIMO can be substituted for others that target the same pathway.

a-e) Flow cytometry density plots of *T*-Venus and SSEA-3 expression in different conditions. **a)** In standard E8 conditions. **b)** Using PRIMO Plus formulation, IWP2 was replaced for DKK1 at 100ng/mL, density plot reveals high double expression 4 days after the first passage. **c)** CHIR99021 was replaced with SB216763 at 10µM, density plot reveals high double expression after 3 days of induction. **d)** LPA was replaced with S1P, density plots demonstrate the ability of S1P to block differentiation, 1.92µM S1P maintained a high proportion of double positive cells after 3 days of induction (optimal concentrated highlighted with a red box). **e)** LPA was replaced with GRI977143, density plots demonstrate the ability of GRI to block differentiation, 4µM GRI maintained a high proportion of double positive cells after 3 days of induction (optimal concentrated highlighted with a red box).

SFig 13

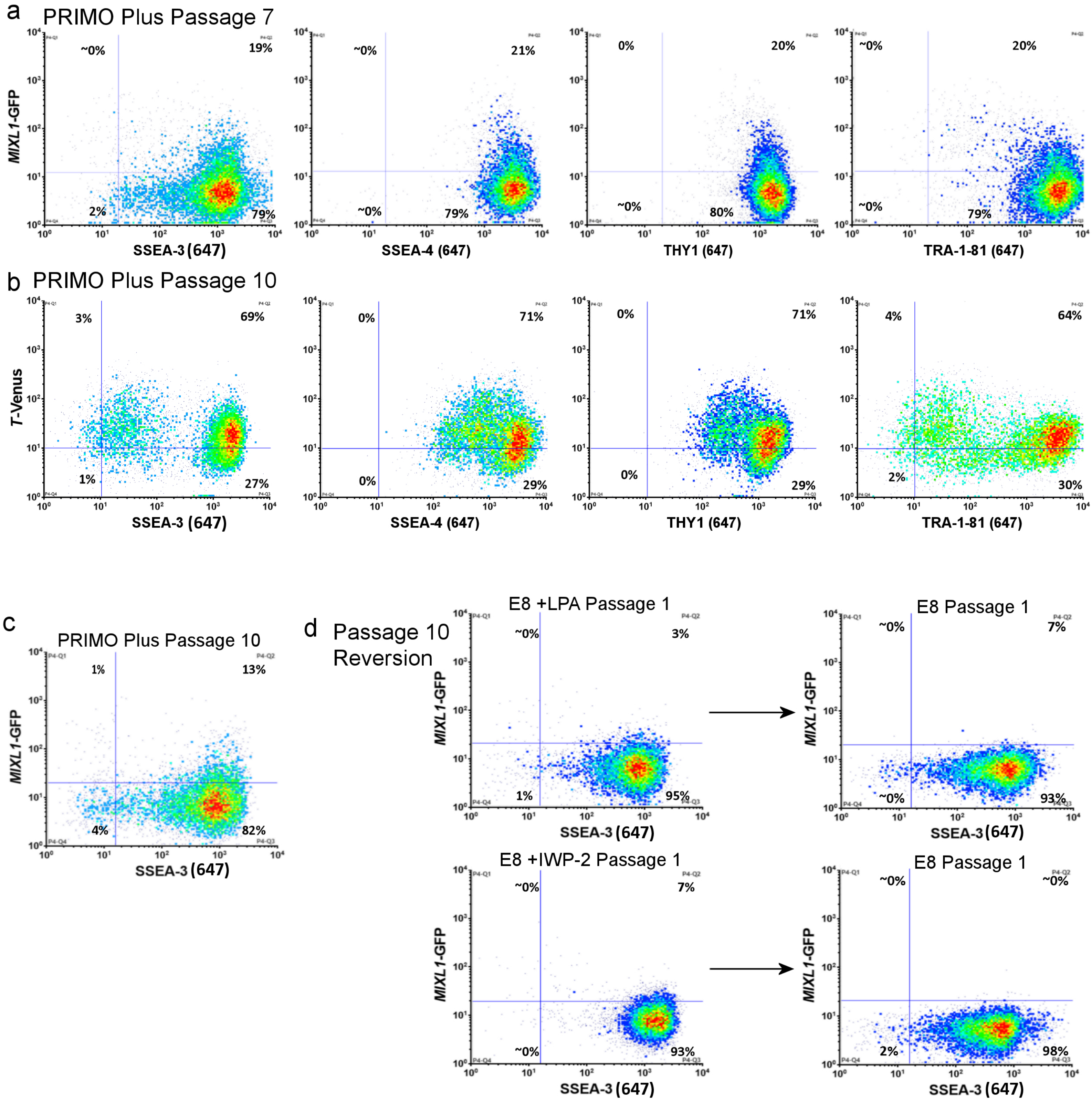
a PRIMO Plus Passage 7
MIXL1-GFP
SSEA-3 (647)
SSEA-4 (647)
THY1 (647)
TRA-1-81 (647)
~0% 19% 2% 79%
~0% 21% ~0% 79%
0% 20% ~0% 80%
~0% 20% ~0% 79%
b PRIMO Plus Passage 10
T-Venus
3% 69% 1% 27%
0% 71% 0% 29%
0% 71% 0% 29%
4% 64% 2% 30%
c PRIMO Plus Passage 10
1% 13% 4% 82%
d Passage 10 Reversion
E8 +LPA Passage 1
~0% 3% 1% 95%
E8 Passage 1
~0% 7% ~0% 93%
E8 +IWP-2 Passage 1
~0% 7% ~0% 93%
E8 Passage 1
~0% ~0% 2% 98%

Supplementary figure 13: Pluripotency-associated Surface Marker Expression of Cells Growing in PRIMO Plus and transitioned back into E8.

a-b) Flow cytometry density plots of *MIXL1*-GFP(**a**) or *T*-Venus(**b**) versus a given pluripotency-associated surface marker at passage 7 (**a**) or 10(**b**) in PRIMO Plus. Markers analysed are SSEA-3, SSEA-4, THY1 and TRA-1-81. Cells show high expression of all markers analysed and a *MIXL1*-GFP or *T*-Venus positive population present. **c)** Flow cytometry density plots of *MIXL1*-GFP and SSEA-3 at passage 10 in PRIMO Plus. **d)** Cells at passage 10 (after 9 passages in PRIMO Plus) were reverted into E8 conditions over two passages with LPA or IWP-2 added to aid reversion in the first passage. Flow cytometry density plots of *MIXL1*-GFP versus SSEA-3 expression are presented for cells grown in those conditions.

SFig 14

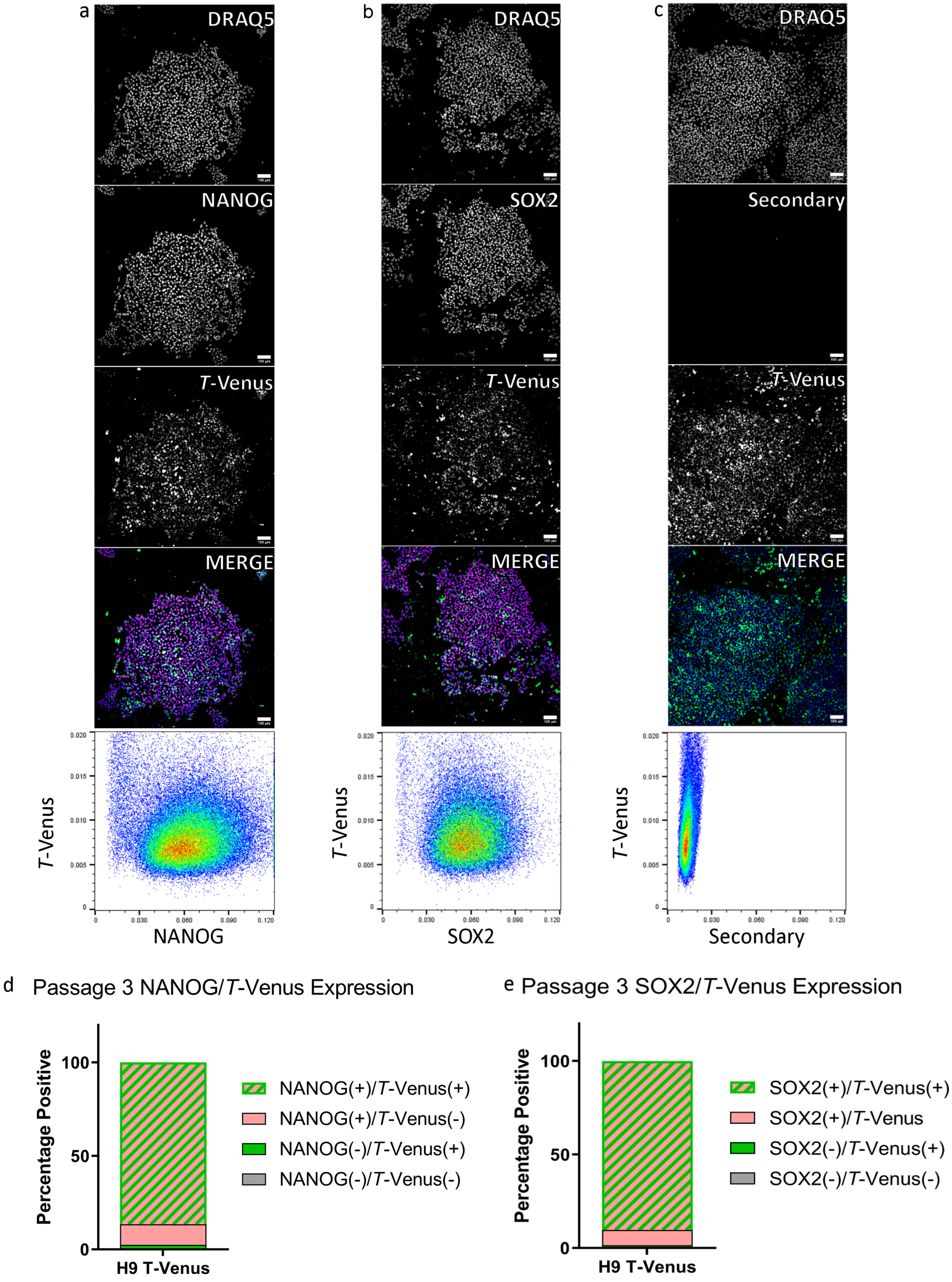
a
b
c
DRAQ5
NANOG
T-Venus
MERGE
SOX2
Secondary
T-Venus
NANOG
SOX2
Secondary
0.020
0.015
0.010
0.005
0
0.030
0.060
0.090
0.120
d
Passage 3 NANOG/T-Venus Expression
Percentage Positive
100
50
0
H9 T-Venus
NANOG(+)/T-Venus(+)
NANOG(+)/T-Venus(-)
NANOG(-)/T-Venus(+)
NANOG(-)/T-Venus(-)
e
Passage 3 SOX2/T-Venus Expression
SOX2(+)/T-Venus(+)
SOX2(+)/T-Venus
SOX2(-)/T-Venus(+)
SOX2(-)/T-Venus(-)

Supplementary figure 14: H9 *T*-Venus show high co-expression of *T*-Venus with NANOG and SOX2.

Immunofluorescence analysis of DRAQ5, *T*-Venus, and **a)** NANOG, **b)** SOX2 expression or **c)** Secondary antibody only staining of H9 *T*-Venus cells in PRIMO Plus. A merged image of all three channels is present below DRAQ5 (Blue), *T*-Venus (Green) and NANOG or SOX2 (Red) (Scale Bar = 100µM). Density plots below images display single cell mean intensity of *T*-Venus versus stained antibody. **d-e)** Stacked percentage bar charts for *T*-Venus, and **d)** NANOG (Bars are mean, n= 3 technical repeats, ~100,000 cells analysed) or **e)** SOX2 (Bars are mean, n= 2 technical repeats, ~100,000 cells analysed) expression grown in PRIMO Plus after three passages. Source data are provided as a Source Data file.

SFig 15

PRIMO Plus 10 passages

HES3 *MIXL1*-GFP

a

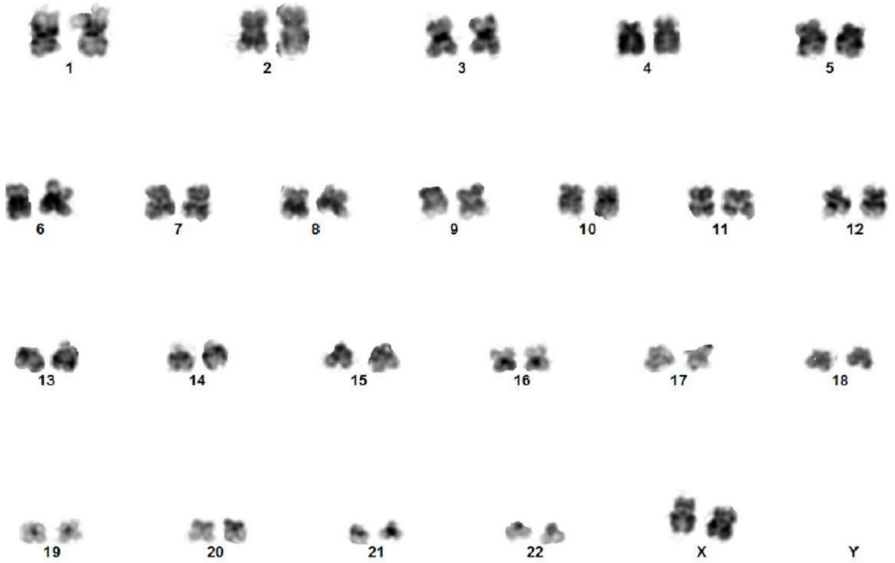

46, XX [30]

H9 *T*-Venus

b

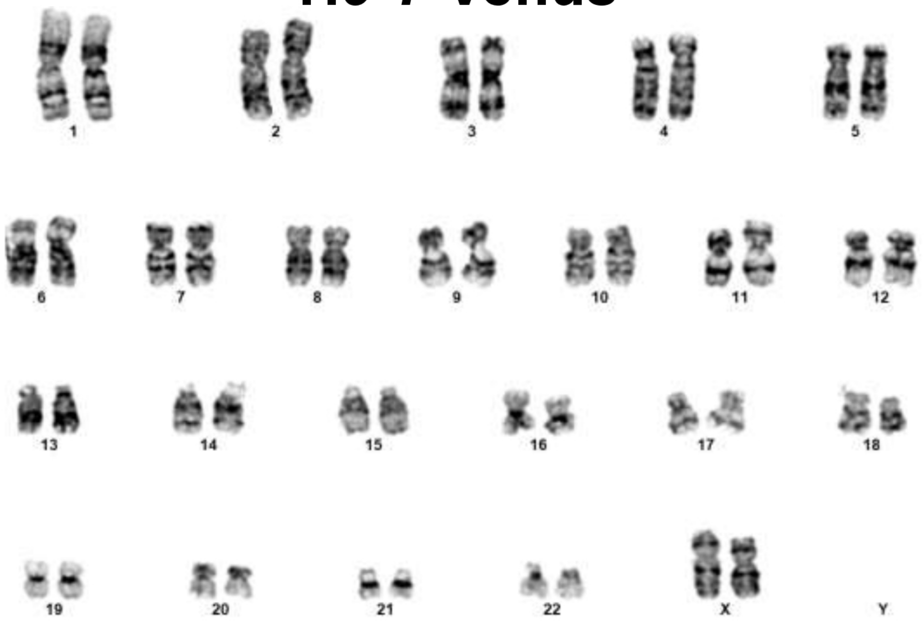

46, XX [30]

Supplementary figure 15: Lines Grown in PRIMO Conditions retain a normal karyotype

Representative images of G-banded metaphase spreads for A) HES3 *MIXL1*-GFP and B) H9 *T*-Venus at at 10 passages in PRIMO PLUS. Both lines display a normal karyotype 46, XX from 30 metaphase spreads with a 95% chance of detecting a 10% population.

SFig 16

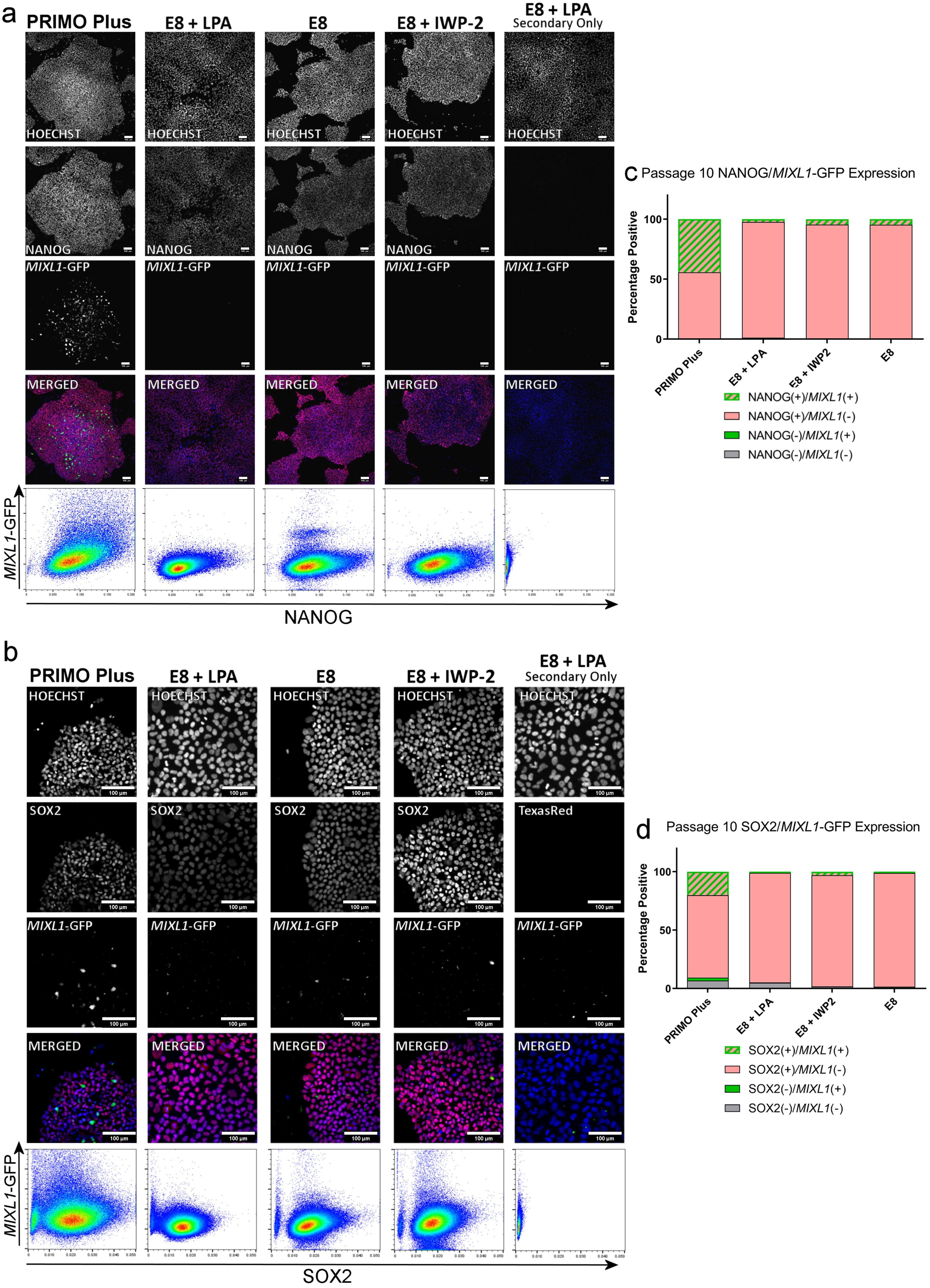

a
PRIMO Plus
E8 + LPA
E8
E8 + IWP-2
E8 + LPA
Secondary Only
HOECHST
NANOG
MIXL1-GFP
MERGED
MIXL1-GFP
NANOG
b
PRIMO Plus
E8 + LPA
E8
E8 + IWP-2
E8 + LPA
Secondary Only
HOECHST
SOX2
TexasRed
MIXL1-GFP
MERGED
100 µm
MIXL1-GFP
SOX2
c Passage 10 NANOG/MIXL1-GFP Expression
Percentage Positive
0
50
100
PRIMO Plus
E8 + LPA
E8 + IWP2
E8
NANOG(+)/MIXL1(+)
NANOG(+)/MIXL1(-)
NANOG(-)/MIXL1(+)
NANOG(-)/MIXL1(-)
d Passage 10 SOX2/MIXL1-GFP Expression
Percentage Positive
0
50
100
PRIMO Plus
E8 + LPA
E8 + IWP2
E8
SOX2(+)/MIXL1(+)
SOX2(+)/MIXL1(-)
SOX2(-)/MIXL1(+)
SOX2(-)/MIXL1(-)

Supplementary figure 16: Passage 10 NANOG and SOX2 expression Analysis

Cells in all conditions at the tenth passage show high expression of NANOG and SOX2. **a-b)** Immunofluorescence analysis of Hoechst, *MIXL1*-GFP, and **a)** NANOG or **b)** SOX2 expression of HES3 *MIXL1*-GFP cells in PRIMO Plus, E8+LPA (0.96µM), E8 alone, E8 with 1µM IWP-2 added and E8+LPA (0.96µM) (Secondary antibody only staining) for 3 days post to 9 passages in PRIMO Plus. A merged image of all three channels is present below Hoechst (Blue), *MIXL1*-GFP (Green) and NANOG or SOX2 (Red) (Scale bar = 100µM). Density plots below images display single cell mean intensity of *MIXL1*-GFP versus stained antibody. **c-d)** Stacked percentage bar charts displaying cell profiler analysis of 3 wells for each condition (Bars are mean, n= 3 technical repeats ~100,000 cells analysed) for *MIXL1*-GFP, and **c)** NANOG or **d)** SOX2 expression grown in PRIMO Plus, E8+LPA (0.96µM), E8 alone and E8 with 1µM IWP-2 added, for 3 days post to 9 passages in PRIMO Plus. Source data are provided as a Source Data file.

Supplementary figure 17: Passage 10 Growth Analysis

SFig 17

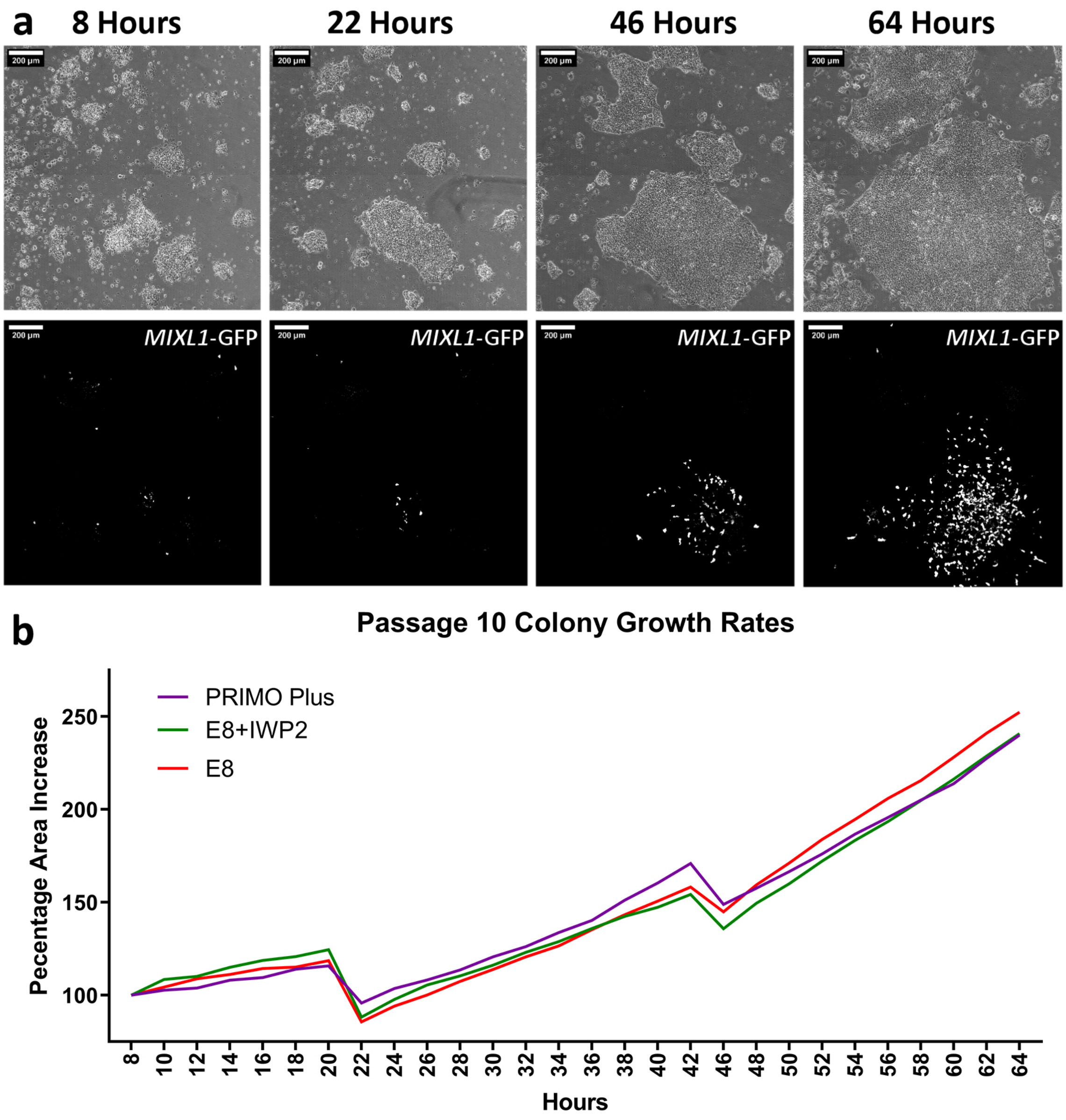
a
8 Hours
22 Hours
46 Hours
64 Hours
200 µm
MIXL1-GFP
b
Passage 10 Colony Growth Rates
PRIMO Plus
E8+IWP2
E8
Pecentage Area Increase
250
200
150
100
8
10
12
14
16
18
20
22
24
26
28
30
32
34
36
38
40
42
46
48
50
52
54
56
58
60
62
64
Hours

a) Phase contrast and green fluorescent images of HES3 *MIXL1*-GFP growing in PRIMO Plus conditions after the tenth passage in this condition. Cells were imaged every 2 hours between 8 and 64 hours post plating (Scale Bar = 200µM). **b)** The graph displays colony growth rates, calculated as percentage covered increase, for cells growing in PRIMO Plus, E8 and E8 with 1µM IWP-2 post 9 passages in PRIMO Plus (n=3 technical replicates, Bars are mean). Cells were imaged using a Nikon Biostation and growth rates quantified by CL-Quant v3.10. Dips in graphs are the result of changing of medium removing cells and debris. Source data are provided as a Source Data file.

SFig 18

a HES3 *MIXL1*-GFP

Passage 4 Reversion

b H9 *T*-VENUS

Passage 4 Reversion

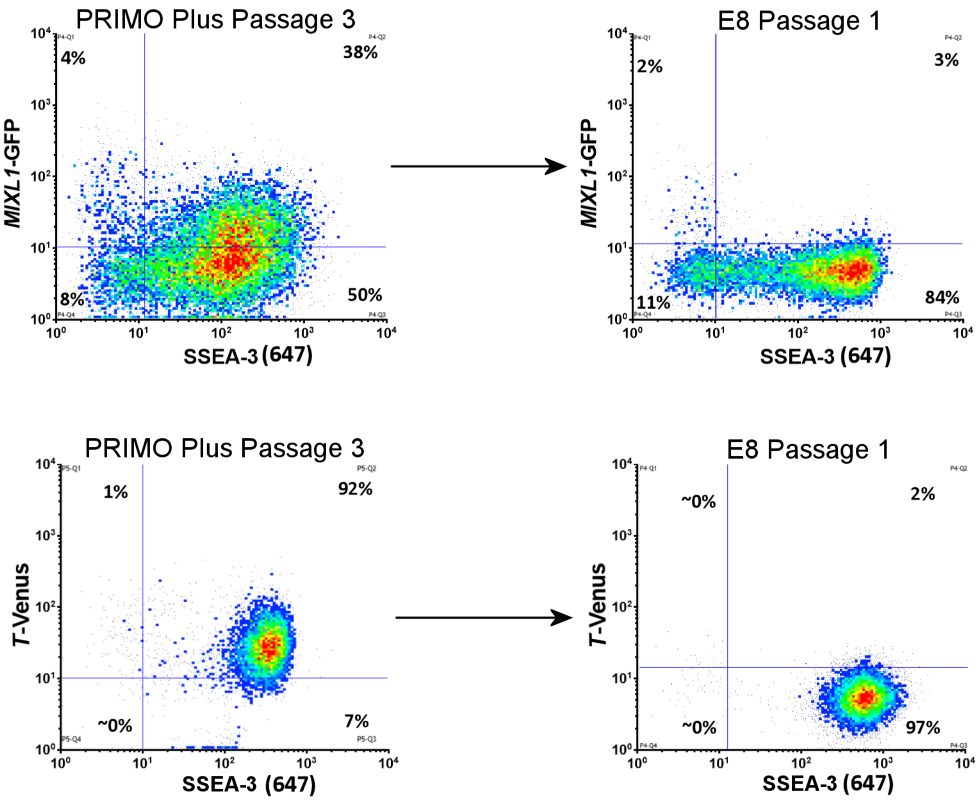

Supplementary figure 18: Pluripotency-associated Surface Marker Expression of Cells Growing in PRIMO Plus and transitioned back into E8.

a-b) Flow cytometry density plots of *MIXL1*-GFP(**a**) or *T*-Venus(**b**) versus SSEA-3 after three passages in PRIMO Plus and then after transitioning into E8. SSEA-3 expression was maintained and reporter gene expression was substantially reduced.

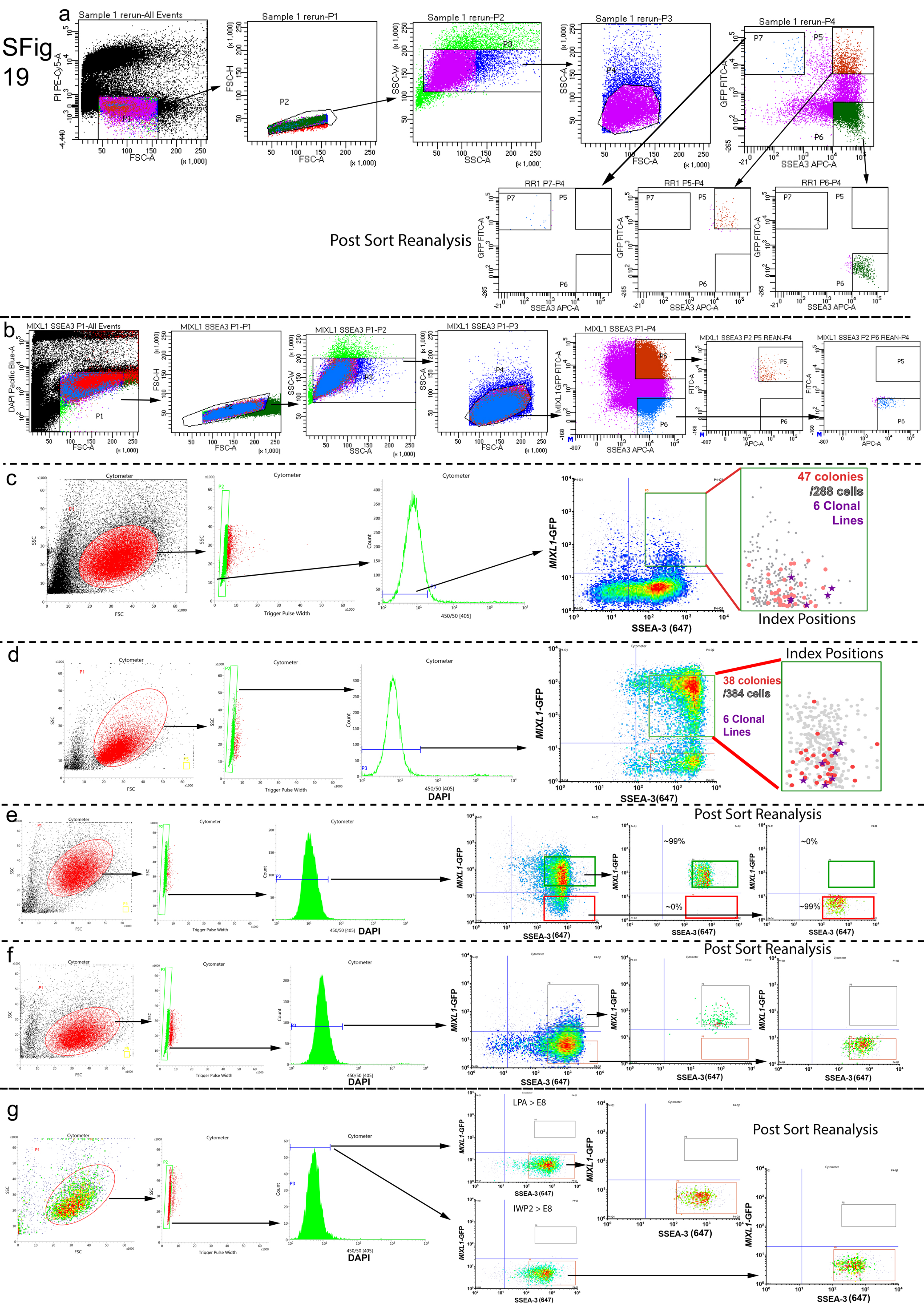

SFig 19
a
Sample 1 rerun-All Events
Sample 1 rerun-P1
Sample 1 rerun-P2
Sample 1 rerun-P3
Sample 1 rerun-P4
Post Sort Reanalysis
RR1 P7-P4
RR1 P5-P4
RR1 P6-P4
b
MIXL1 SSEA3 P1-All Events
MIXL1 SSEA3 P1-P1
MIXL1 SSEA3 P1-P2
MIXL1 SSEA3 P1-P3
MIXL1 SSEA3 P1-P4
MIXL1 SSEA3 P2 P5 REAN-P4
MIXL1 SSEA3 P2 P6 REAN-P4
c
47 colonies
/288 cells
6 Clonal Lines
Index Positions
d
Index Positions
38 colonies
/384 cells
6 Clonal Lines
e
Post Sort Reanalysis
~99%
~0%
~0%
~99%
f
Post Sort Reanalysis
g
LPA > E8
IWP2 > E8
Post Sort Reanalysis

Supplementary figure 19: Fluorescence activated cell sorting (FACS) gating strategy.

a-g) Displays the gating strategy for the FACS performed in this manuscript. All sorts were performed on HES3 *MIXL1*-GFP cells stained for SSEA-3 primary and Alexafluor 647 goat anti mouse secondary antibodies. Negative gates were set using wildtype HES3 stained with P3X and Alexafluor 647 goat anti mouse secondary, detailed in the methods section and source data. Panels **a)** and **b)** were performed on the BD Facs Aria III, using FACS Diva software, the remaining panels **c-g)** were performed on the BD FacsJAZZ, using BD Sortware software. **a)** and **b)** For Live/Dead discrimination scatter plot of Forward Scatter (FSC) versus **a)** Propidium Iodide-Cy5 or **b)** DAPI Pacific Blue emission P1 gate identifies the live cell population. Doublet discrimination scatter plots of Forward Scatter (FSC) and Side scatter (SSC) variables decipher the single cell population from cells running through the flow cytometer. The FITC and APC channels were used to assess the expression of *MIXL1*-GFP and SSEA-3, respectively. **a)** relates to Figure 1. **b)** relates to Figure 4a,b,d,e and f. **c-g)** A scatter plot of Forward Scatter (FSC) versus Side scatter (SSC) is used to set the P1 gate in red, which identifies the cell population. Doublet discrimination is performed using Trigger Pulse width versus SSC, P2 in green identifies single cells. A histogram plot was used for Live/Dead discrimination. DAPI staining is analysed with excitation by the violet laser 405nm and emission filter 450/50. P3 in blue identifies live cells. Live cells were then assessed on density plots of HES3 *MIXL1*-GFP (488nm excitation and emission filter 530/40) stained for SSEA-3 (640 excitation and emission filter 660/20). Sorting gates are shown as rectangles on the plots. **c)** and **d)** single cell cloning experiments and the index position of the sorted cells is shown. **e)** bulk sorts for EB differentiations and post sort analysis is shows good separation of *MIXL1*-GFP(+) and (-) cells**. f)** and **g)** bulk sorts and post sort reanalysis is shown for samples immediately before sorting the same fractions directly into Trizol for RNA-sequencing. **c)** relates to Figure 2a. **d)** relates to Figure 5b. **e)** relates to Figure 5d. **f)** depicts the sorting of HES3 *MIXL1* cells at passage 10 of PRIMO

Plus culturing relating to Figure 7e-f. **g)** depicts the sorting of HES3 *MIXL1* reverted cells, reverted with LPA or IWP-2 present, relating to Figure 7e-f.

Supplementary Table 1 Single Cell qPCR assay list

Assay ID	Gene Symbol	RefSeq	Amplicon Length	Detects gDNA	Best Coverage
**Hs01060665_g1**	*ACTB*	NM_001101.3	63	Yes	Yes
**Hs00154192_m1**	*BMP2*	NM_001200.2	60	No	Yes
**Hs03676628_s1**	*BMP4*	NM_130850.2;NM_001202.3;NM_130851.2	116	Yes	Yes
**Hs01034913_g1**	*BMPR1A*	NM_004329.2	94	Yes	Yes
**Hs00193796_m1**	*CER1*	NM_005454.2	92	No	Yes
**Hs01897804_s1**	*CITED2*	NM_001168388.2;NM_001168389.2;NM_006079.4	106	Yes	Yes
**Hs00607528_s1**	*CLDN6*	NM_021195.4	154	Yes	Yes
**Hs00164004_m1**	*COL1A1*	NM_000088.3	66	No	Yes
**Hs00976734_m1**	*CXCR4*	NM_003467.2;NM_001008540.1	153	No	No
**Hs00171876_m1**	*DNMT3B*	NM_001207055.1;NM_001207056.1;NM_175848.1;NM_175849.1;NM_175850.2;NM_006892.3	55	No	Yes
**Hs00172872_m1**	*EOMES*	NM_001278183.1;NM_001278182.1;NM_005442.3	81	No	Yes
**Hs01549976_m1**	*FN1*	NM_212482.1;NM_054034.2;NM_002026.2;NM_212478.1;NM_212474.1;NM_212476.1	81	No	Yes
**Hs00232764_m1**	*FOXA2*	NM_021784.4;NM_153675.2	66	No	Yes
**Hs00255287_s1**	*FOXD3*	NM_012183.2	78	Yes	Yes
**Hs00173503_m1**	*FRZB*	NM_001463.3	108	No	Yes
**Hs00246256_m1**	*FST*	NM_006350.3;NM_013409.2	108	No	No
**Hs00544355_m1**	*GAL*	NM_015973.3	125	No	Yes
**Hs00171403_m1**	*GATA4*	NM_002052.3	68	No	Yes
**Hs00232018_m1**	*GATA6*	NM_005257.4	91	No	Yes
**Hs00906630_g1**	*GSC*	NM_173849.2	100	No	No
**Hs00193435_m1**	*HAS2*	NM_005328.2	63	No	Yes
**Hs00242160_m1**	*HHEX*	NM_002729.4	110	Yes	Yes
**Hs00705137_s1**	*IFITM1*	NM_003641.3	93	Yes	Yes
**Hs01547673_m1**	*ITGA5*	NM_002205.2	54	No	Yes
**Hs00761767_s1**	*KRT19*	NM_002276.4	116	Yes	Yes
**Hs00764128_s1**	*LEFTY1*	NM_020997.3	136	Yes	Yes
**Hs00745761_s1**	*LEFTY2*	NM_001172425.1;NM_003240.3	102	Yes	Yes
**Hs00355202_m1**	*LGALS1*	NM_002305.3	63	No	Yes
**Hs00232144_m1**	*LHX1*	NM_005568.3	60	No	Yes
**Hs00702808_s1**	*LIN28A*	NM_024674.4	143	Yes	Yes
**Hs00430824_g1**	*MIXL1*	NM_031944.1	152	No	No

Assay ID	Gene Symbol	RefSeq	Amplicon Length	Detects gDNA	Best Coverage
**Hs00899658_m1**	*MMP1*	NM_001145938.1;NM_002421.3	64	No	Yes
**Hs01548727_m1**	*MMP2*	NM_004530.4;NM_001127891.1	65	No	Yes
**Hs01085598_g1**	*MYL7*	NM_021223.2	74	No	Yes
**Hs04399610_g1**	*NANOG*	NM_024865.2	101	Yes	No
**Hs00378379_m1**	*NCLN*	NM_020170.3	65	No	Yes
**Hs00415443_m1**	*NODAL*	NM_018055.4	68	No	Yes
**Hs00219496_m1**	*PAF1*	NM_019088.3;NM_001256826.1	100	No	Yes
**Hs04260367_gH**	*POU5F1*	NM_001173531.1;NM_002701.4;NM_203289.4	77	Yes	Yes
**Hs01375212_g1**	*RPS18*	NM_022551.2	93	Yes	Yes
**Hs00183425_m1**	*SMAD2*	NM_001135937.2;NM_001003652.3;NM_005901.5	129	No	No
**Hs00195591_m1**	*SNAI1*	NM_005985.3	66	Yes	Yes
**Hs00751752_s1**	*SOX17*	NM_022454.3	149	Yes	Yes
**Hs01053049_s1**	*SOX2*	NM_003106.3	91	Yes	Yes
**Hs00610080_m1**	*T*	NM_001270484.1;NM_003181.3	132	No	Yes
**Hs00761239_s1**	*TAGLN2*	NM_001277224.1;NM_001277223.1;NM_003564.2	163	No	Yes
**Hs02339499_g1**	*TDGF1*	NM_003212.3;NM_001174136.1	170	Yes	No
**Hs00902257_m1**	*WNT3*	NM_030753.4	76	No	Yes
