# Supplementary material for: Generation and trapping of a mesoderm biased state of human pluripotency
Source: Nat Commun. 2020 Oct 5;11:4989. doi: 10.1038/s41467-020-18727-8 (PMC7536399; doi:10.1038/s41467-020-18727-8)
Supplement: Supplementary file 3 — Reporting Summary [file 41467_2020_18727_MOESM3_ESM.pdf]

nature research

Corresponding author(s): Peter Andrews, Dylan Stavish

Last updated by author(s): Aug 18, 2020

Reporting Summary

Nature Research wishes to improve the reproducibility of the work that we publish. This form provides structure for consistency and transparency in reporting. For further information on Nature Research policies, see our Editorial Policies and the Editorial Policy Checklist.

Statistics

For all statistical analyses, confirm that the following items are present in the figure legend, table legend, main text, or Methods section.

n/a	Confirmed	
☐	☒	The exact sample size (*n*) for each experimental group/condition, given as a discrete number and unit of measurement
☒	☐	A statement on whether measurements were taken from distinct samples or whether the same sample was measured repeatedly
☐	☒	The statistical test(s) used AND whether they are one- or two-sided<br>*Only common tests should be described solely by name; describe more complex techniques in the Methods section.*
☒	☐	A description of all covariates tested
☐	☒	A description of any assumptions or corrections, such as tests of normality and adjustment for multiple comparisons
☐	☒	A full description of the statistical parameters including central tendency (e.g. means) or other basic estimates (e.g. regression coefficient) AND variation (e.g. standard deviation) or associated estimates of uncertainty (e.g. confidence intervals)
☐	☒	For null hypothesis testing, the test statistic (e.g. *F*, *t*, *r*) with confidence intervals, effect sizes, degrees of freedom and *P* value noted<br>*Give P values as exact values whenever suitable.*
☒	☐	For Bayesian analysis, information on the choice of priors and Markov chain Monte Carlo settings
☒	☐	For hierarchical and complex designs, identification of the appropriate level for tests and full reporting of outcomes
☒	☐	Estimates of effect sizes (e.g. Cohen's *d*, Pearson's *r*), indicating how they were calculated

Our web collection on statistics for biologists contains articles on many of the points above.

Software and code

Policy information about availability of computer code

Data collection	BD FACS Sortware 1.2.0.142, BD FACS Diva 8.0.1,
Data analysis	Monocle2, SeqMonk 1.46, Cell Profiler 2.2, FlowJo v 10.6, FastQC v 0.11.5, HISAT2 v2.1.0, QoRTs v1.1.8, cufflinks v 2.2.1, DESeq2, ToppGene, ReviGO, Genesis 1.8.1, Thermofisher's hPSC scorecard analysis software, GraphPad Prism v7, CL-quant v3.10

For manuscripts utilizing custom algorithms or software that are central to the research but not yet described in published literature, software must be made available to editors and reviewers. We strongly encourage code deposition in a community repository (e.g. GitHub). See the Nature Research guidelines for submitting code & software for further information.

Data

Policy information about availability of data

All manuscripts must include a data availability statement. This statement should provide the following information, where applicable:

- Accession codes, unique identifiers, or web links for publicly available datasets
- A list of figures that have associated raw data
- A description of any restrictions on data availability

Source data for Figures 1c-e, 2a, 4b, e, 5b, d, 6, and 7b, d, e and Supplementary Figures 1b, c, d, 2, 3c, 4, 5, 9c, e, 11c, 14b, c, 16c, d, and 17b have been provided in a Supplementary Data File. RNA sequencing data that supports the findings of this study have been deposited in the ArrayExpress database at EMBL-EBI under the accession number E-MTAB-9474 (https://www.ebi.ac.uk/arrayexpress/experiments/E-MTAB-9474). All other supporting data is available from the corresponding author upon request.

Field-specific reporting

Please select the one below that is the best fit for your research. If you are not sure, read the appropriate sections before making your selection.

☒ Life sciences ☐ Behavioural & social sciences ☐ Ecological, evolutionary & environmental sciences

For a reference copy of the document with all sections, see nature.com/documents/nr-reporting-summary-flat.pdf

Life sciences study design

All studies must disclose on these points even when the disclosure is negative.

Sample size	No statistical methods were used for sample size calculation. For single cell qPCR, 80 cells per fraction was the largest size that was feasible within the experimental budget. Establishing only six clonal lines from cloning experiments was determined as the largest size that was feasible. Two ES cell lines carrying mesoderm-associated gene reporters, HES3-MIXL1 and H9 T-Venus, were used as they could indicate early lineage priming and an iPS line, MIFF1 was used to confirm results in an induced pluripotent line.
Data exclusions	In our single cell qPCR experiments 8 of our 320 single cells were omitted from the analysis. These cells were removed due to very low to no detectable expression of the housekeeping genes ACTB and RPS18.
Replication	Where possible experiments were carried out on at least two independent cell lines and sometimes three independent cell lines. Work using clonal lines were conducted on 3-6 independent clones.
Randomization	Randomization was not possible for the experiments detailed here.
Blinding	N/A

Reporting for specific materials, systems and methods

We require information from authors about some types of materials, experimental systems and methods used in many studies. Here, indicate whether each material, system or method listed is relevant to your study. If you are not sure if a list item applies to your research, read the appropriate section before selecting a response.

Materials & experimental systems

n/a	Involved in the study	
☐	☒	Antibodies
☐	☒	Eukaryotic cell lines
☒	☐	Palaeontology and archaeology
☒	☐	Animals and other organisms
☒	☐	Human research participants
☒	☐	Clinical data
☒	☐	Dual use research of concern

Methods

n/a	Involved in the study	
☒	☐	ChIP-seq
☐	☒	Flow cytometry
☒	☐	MRI-based neuroimaging

Antibodies

Antibodies used	Primary antibodies P3X (1:10), BF4(1:10), CD9(CH8)(1:10), SSEA-3(1:10), SSEA-4(1:100), THY1(CD90)(1:10), TRA-1-60(1:20), TRA-1-81 (1:20) and TRA-2-49(1:10) were all prepared in house from the relevant hybridomas. Anti-NANOG (XP® #4903, Cell Signalling Technology, 1:400), ANTI-SOX2 (XP® #3579, Cell Signalling Technology, 1:400). Anti-SOX10 (Cell Signalling Technology, 89356S, 1:500).<br>Secondary antibodies: Goat anti Mouse Affinipure IgG+IgM (H+L) AlexaFluor 647 (Stratech (Jackson ImmunoResearch) 115-605-044-JIR, Polyclonal 1:200), Goat anti Rabbit Affinipure IgG (H+L) AlexaFluor 594 (Stratech (Jackson ImmunoResearch) 111-585-003-JIR, Polyclonal 1:200)<br>Conjugates CD147 APC-conjugated antibody (R&D Systems, FAB3195A, Monoclonal Mouse IgG1 Clone # TRA-1-85, 1:100), CD43 PE-conjugated antibody (Biolegend, 343203, Mouse monoclonal Clone # CD43-10G7, 1:100) and CD34 PECy7-conjugated antibody (Biolegend 343516, Mouse monoclonal Clone #581, 1:160).
Validation	In-House antibodies were titrated against the pluripotent embryonal carcinoma line, NTERA2, to validate reactivity or non-reactivity for P3X. Anti-NANOG and Anti-SOX2 have been validated against NTERA2 by the manufacturers and were titrated in our hands against a human embryonic stem cell line grown in self-renewal conditions. The threshold for positive expression was set with staining of either P3X and secondary antibody or secondary antibody alone.<br>CD147 APC-conjugated antibody: "Detects human TRA-1-85 antigen in flow cytometry" verified by positive staining human peripheral blood lymphocytes and monocytes by the manufacturer.<br>CD43 PE-conjugated antibody: "Each lot of this antibody is quality control tested by immunofluorescent staining with flow cytometric analysis" verified by positive staining human peripheral blood lymphocytes by the manufacturer.<br>CD34 PECy7-conjugated antibody: "Each lot of this antibody is quality control tested by immunofluorescent staining with flow

cytometric analysis" verified by positive staining human peripheral blood by the manufacturer.

Eukaryotic cell lines

Policy information about cell lines

Cell line source(s)	Four human pluripotent stem cell lines were used in this project, Embryonic Stem cell lines, HES3 (Cooper et al., 2002), HES3-MIXL1 (Davis et al., 2008) and H9 T-Venus (Mendjan et al., 2014), and induced pluripotent stem cell line, MIFF1 (Desmarais et al., 2016). Andrew Elefanty (Monash University, Australia) provided HES3 and HES3-MIXL1 lines, Roger Pedersen and Daniel Ortmann of (Cambridge University, UK) provided H9 T-Venus line, MIFF1 was generated in-house (University of Sheffield) by Christian Unger by mRNA reprogramming of CRL-2429 Human fetal foreskin fibroblasts (ATCC, Manassas, VA, USA).
Authentication	Not authenicated
Mycoplasma contamination	Not tested
Commonly misidentified lines (See ICLAC register)	No commonly misidentified lines were used

Flow Cytometry

Plots

Confirm that:

- [x] The axis labels state the marker and fluorochrome used (e.g. CD4-FITC).
- [x] The axis scales are clearly visible. Include numbers along axes only for bottom left plot of group (a 'group' is an analysis of identical markers).
- [x] All plots are contour plots with outliers or pseudocolor plots.
- [x] A numerical value for number of cells or percentage (with statistics) is provided.

Methodology

Sample preparation	When preparing single cells for the flow cytometry, media was aspirated from the flask and cells were washed once with PBS. Cells were then treated with either 0.25% Trypsin in EDTA for 1-2 minutes, TrypLE Select (Thermo Fisher Scientific, 12563029) for 5 minutes or Accutase (Thermofisher, #A1110501) for 10 mins, all at 37°C. Media was added to the flasks, if using Trypsin or TrypLE media contained 10% FCS to neutralise the trypsin, when using accutase, standard growth media was used as accutase is neutralised by dilution. Cells were counted using a haemocytometer. Media containing cells was then centrifuged at 1000rpm for 3mins to pellet cells. Cells were resuspended in DMEM (without phenol red) and 10% FCS at a density of 1x107 per mL. 100µL of the samples was dispensed into 5ml tubes and antibodies were added at the appropriate dilution. After addition of the primary antibody or conjugates, cells were incubated at 4°Cfor 30 minutes. Cells were then washed with DMEM/FCS and centrifuged at 1000 rpm for 3 minutes. After pelleting, the cells are resuspended in 200µL of DMEM/FCS. Secondary antibody was added when required at the appropriate dilutions and incubated at 4°C for 30 minutes. Cells were then washed with DMEM/FCS, centrifuged for 3 minutes at 1000rpm and resuspended in fresh DMEM/FCS for analysis by flow cytometry. DAPI (ThermoFisher, #62248), Hoechst 33258 or Prodidium Iodide was added at 1:10,000 and used for live/dead discrimination.
Instrument	BD FACS JAZZ and BD FACS ARIA III
Software	Sortware for BD FACS Jazz or FACS Diva for BD FACS Aria III. Some data analysis and plots were generated using FlowJo version 10
Cell population abundance	For cell sorting gates were positioned to allow a suitable margin between to populations to ensure accurate separation. Cells were sorted into the appropriate vessels and post sort the samples were reanalysed on the flow cytometer. Only samples that had high efficiency percentages, >90%, were used in further experiments, a representative image is in the supplementary data. For experiments such as for RNA-sequencing cells were deposited straight into lysis buffer, precluding their reanalysis. For these experiments equivalent numbers of cells were sorted immediately before into PBS(w/o Ca+, Mg++) and reanalysed to estimate the sorting efficiency of the subsequent samples sorted into lysis buffer. To ensure accurate deposition of single cells into individual wells of the 96 well plate for cloning or single cell qPCR, firstly empty droplets were sorted to align the stream to the middle of the wells. After this, we used a mixture of Red (Accudrop) and Green (Big Bang) fluorescent beads to determine whether the sorter was separating populations accurately. Beads were gated using the BD Sortware program then a row of Red beads and row of Green beads were sorted as single beads. This was performed as an index sort just as the subsequent cell sorts would be. 96 well plates were then imaged on the InCell Analyzer at 4x with both the FITC and Cy5 channels to verify the correct number, colour and placement of the deposited beads was correct, a representative image is in the supplementary data.
Gating strategy	For the BD FACS Jazz: The initial cell population was identified on a scatter plot of Forward Scatter (FSC) versus Side scatter (SSC) from cells running through the flow cytometer, a gate was drawn around the compact population. Doublets were discriminated using a scatter plot of trigger Pulse width versus SSC. Live/ Dead discrimination was set using a histogram plot

or scatter plot of DAPI intensity. To set baselines for MIXL1-GFP and negative secondary 647, unlabelled HES3 line was harvested and stained for P3X or an isotype control. P3X is an IgG1 antibody which is secreted from the parent myeloma which all in house antigens were derived. P3X shows minimal reactivity to human cells (Kohler and Milstein, 1975). Positive gates were set according to HES3 P3X negative controls. Samples were also stained for P3X to assess non-specific binding. All flow cytometry analysis contained P3X samples for baseline setting. An example of the gating strategy is in the supplementary data.

For the FACS Aria III, the Live/dead discrimination was performed first using DAPI, PI or Hoechst versus FSC-A. Doublet discrimination was perfomed by plotting FSC-H versus FSH-A, SSC-W versus SSC-A and FSC-A versus SSC-A to identify the single cell population. The remaining baseline was set as above using unlabeled HES3 stained for P3X. An example of the gating strategy is in the supplementary data.

☒ Tick this box to confirm that a figure exemplifying the gating strategy is provided in the Supplementary Information.
